# Supplementary material for: Integrated genomic analysis reveals key features of long undecoded transcript isoform-based gene repression
Source: Mol Cell. 2021 May 20;81(10):2231–2245.e11. doi: 10.1016/j.molcel.2021.03.013 (PMC8153250; doi:10.1016/j.molcel.2021.03.013)
Supplement: Document S1. Figures S1–S7 and Tables S5 and S7 [file mmc1.pdf]

Molecular Cell, Volume *81*

Supplemental information

Integrated genomic analysis reveals key features of long undecoded transcript isoform-based gene repression

Amy Tresenrider, Kaitlin Morse, Victoria Jorgensen, Minghao Chia, Hanna Liao, Folkert Jacobus van Werven, and Elçin Ünal

Supplemental Figure Legends:

Figure S1. TL-seq and direct RNA sequencing for a LUTI gene and a meiosis-specific gene, Related to Figure 1

A. Genome browser views of TL-seq and direct RNA sequencing using Nanopore at the *NDC80* locus. The TL-seq and Direct RNA sequencing were performed in cells with the *pCUP-IME1/pCUP-IME4* meiotic induction system (*UB14584*). The premeiotic time point was taken after 2 hours in SPO, and the meiotic prophase time point was taken after 4 hours in SPO, which corresponds to 2 hours after 50 μM $CuSO_4$ addition. **B.** As in **(A)** but for *SPO11*.

Figure S2. Ume6 enrichment for the genes without 5' extensions and RNA-seq browser from *UME6* and *ume6* T99N strains, Related to Figure 1

A. Heatmap of Ume6 enrichment over input for all of the genes without 5'-extensions (n=5298). A representative image from one of three replicates is shown. **B-C.** Genome browser views of RNA-seq at the **(B)** *NDC80* and the (**C**) *APL4 locus.* RNA-seq was performed on strains expressing either WT Ume6 (*UB20649*) or Ume6 with a T99N mutation (*UB22629*). The strains also harbor the *pCUP1-IME1/pCUP1-IME4* meiotic induction system and were induced with 50 μM $CuSO_4$ at 2 hours in sporulation medium (SPO). Cells were collected after 2 or 4 hours in SPO.

Figure S3. Contribution of LUTI and PROX isoforms to total RNA abundance and assessment of RNA-seq similarity between this study and Cheng et al. 2018, Related to Figure 2

A. Scatterplots comparing RNA-seq and TL-seq. Cells harboring the *pCUP1-IME1/pCUP1-IME4* meiotic induction system and were induced with 50 μM $CuSO_4$ at 2 hours in sporulation medium (SPO). Analysis was performed with data from cells collected during meiotic prophase (4 hours in SPO). In all plots the teal data points display RNA-seq vs TL-seq transcripts per million for genes without 5'-extensions. All orange points indicate loci with 5'-extended transcripts. TL-seq values for genes with 5'-extended transcripts were calculated either from PROX-specific TPM (Left), LUTI-specific TPM (Middle), or the addition of both PROX- and LUTI-specific TPM (Right). The Spearman's rank correlation coefficient (ρ) was calculated and is displayed on the upper left corner for both teal and orange points separately. **B.** Scatterplots comparing RNA-seq performed in this study to RNA-seq performed in Cheng et al. 2018. In this study, cells with the *pCUP1-IME1/pCUP1-IME4* meiotic induction system (*UB14584*) were collected for RNA-seq (in duplicate) after 4 hours in sporulation medium (SPO), which corresponds to 2 hours after induction of meiosis by 50 μM $CuSO_4$. In Cheng et al., a prototrophic strain was used. Cells were collected after 1.5 (left), 3 (middle) or 4.5 (right) hours in SPO. The Spearman's rank correlation coefficient (ρ) was calculated and is displayed on the upper left corner. **C.** GSEA enrichment plot for the SC/Recombination gene set defined in Brar et al. (2012). Analysis was performed with RNA-seq (duplicates) collected from cells harboring the*pCUP1-IME1/pCUP1-IME4*

meiotic induction system (*UB14584*). Enrichment was determined by comparing gene expression at 4 hours in SPO (meiotic prophase) to 2 hours (premeiotic).

Figure S4. The effect of uORF deletion or *upf1Δ* on LUTI abundance and protein level, Related to Figure 3

A. Cells with either wild-type uORFs or uORFs with ATGs mutated to ATC and a 3V5 tagged *APL4* (*WT: UB18539, uORF*Δ: *UB26122*), *HSP60* (*WT: UB18335, uORFΔ:UB26123*), or *MSC6* (*WT: UB18238, uORFΔ:UB26124*) were induced to undergo meiosis with 50 µM $CuSO_4$ after 2 hours in SPO using the *pCUP1-IME1/pCUP1-IME4* meiotic induction system. Immunoblot was performed against the 3V5 epitope and Hxk2 as a loading control. One of two replicates is displayed. **B-M.** Wild-type or *upf1Δ* strains harboring 3V5 tagged *APL4* (*WT: UB18539, upf1*Δ: *UB26707*), *HSP60* (*WT: UB18335, upf1Δ:UB26705*), or *MSC6* (*WT: UB18238, uORFΔ:UB26708*) were induced to undergo meiosis with 50 µM $CuSO_4$ after 2 hours in SPO using the *pCUP1-IME1/pCUP1-IME4* meiotic induction system. (**B-D**) RNA blots whereby transcripts were detected using a probe directed against the 3V5 epitope and its linker sequence. rRNA was stained by methylene blue as a loading control. (**E-G**) qPCR quantification of LUTI transcripts using the matched RNA from **B-D.** Quantification was performed in reference to the levels of the meiotic housekeeping gene *PFY1*. **(H-J)** Immunoblot with α-3V5 and α-Hxk2 antibodies. (**K-M**) Quantification of immunoblots in **H-J.** Abundance of the 3V5 tagged protein was quantified by first normalizing to the Hxk2 loading control and then to the first time point (0 h).

Figure S5. Single gene analysis of LUTI deletion, Related to Figure 4

A-B. LUTI deletion analysis of additional genes. Cells with the *pCUP1-IME1/pCUP1-IME4* meiotic induction system were induced to enter meiosis with 50 µM $CuSO_4$ after 2 hours in SPO. **A.** Experiments were performed with a *MSC6-3V5* tagged strain harboring either wild type (*UB18238*) or LUTI deletion (*UB18190*). RNA blots and Immunoblots were performed on samples collected between 0-6 hours in SPO. RNA blots were performed with a probe specific for 3V5 and its linker. Methylene blue detection of rRNA bands was the loading control. Immunoblots were performed with a α-V5 antibody to recognize the 3V5-tagged proteins. Hxk2 was used as a loading control. The blots represent one of two replicates. Quantification of the immunoblot is displayed on the right. 3V5 signal was quantified relative to Hxk2 and then relative to 0 hr. Graphs represent one of two replicates **B.** Same as A, but for *APL4-3V5* tagged strains with either wild type (*UB18539*) or LUTI deletion (*UB18181*).

Figure S6. Alterations to chromatin over the PROX promoter upon LUTI expression, Related to Figure 5

A. Metagene analysis of H3K36me3 ChIP-seq for LUTI and non-LUTI genes in both the premeiotic and meiotic prophase stages. The ChIP was performed in cells with the *pCUP1-IME1/pCUP1-IME4* meiotic induction system *(UB14584).* The premeiotic time point was taken after 2 hours in SPO, and the meiotic prophase time point was taken

after 4 hours in SPO, which corresponds to 2 hours after 50 μM $CuSO_4$ addition. The images are from one of three replicates. **B.** Same as (**A**) but for H3K4me2. **C.** Heatmap of H3K36me3 ChIP-seq in premeiotic and meiotic prophase. The ChIP was performed in cells with the *pCUP1-IME1/pCUP1-IME4* meiotic induction system (*UB14584*). The premeiotic time point was taken after 2 hours in SPO, and the early meiotic time point was taken after 4 hours in SPO, which corresponds to 2 hours after 50 μM $CuSO_4$ addition. The plot is centered around the PROX TSS. Top: LUTIs, bottom: canonical transcripts. The images are from of one of three replicates. **D.** Same as **(C)** but for H3K4me2. **E-F.** Genome browser views of MNase-seq and TL-seq. The TL-seq and MNase DNA digestion was performed in cells with the *pCUP-IME1/pCUP-IME4* meiotic induction system (*UB14584*). The premeiotic time point was taken after 2 hours in SPO, and the meiotic prophase time point was taken after 4 hours in SPO, which corresponds to 2 hours after 50 μM $CuSO_4$ addition. Three candidate genes, *RPO41* **(E)**, *APL4* **(F)** and *SWI4* **(G)**, all of which are robustly transcriptionally repressed are shown. The green box denotes the approximate location of the PROX promoter.

Figure S7. Reduction in PROX transcript abundance shows significant correlation with a select number of criteria, Related to Figure 6

Scatterplots of the relationships tested in Figure **6A**. Spearman's correlation coefficients (ρ) are displayed on the upper left corner.

Supplemental Table Legends:

Table S5. Translational efficiency (TE) fold-change between meiotic prophase and premeiotic stage for LUTIs with < 4 ATG codons in the region between the LUTI and the PROX TSSs, Related to Figure 3

Table S7. Primers used in this study, Related to STAR Methods

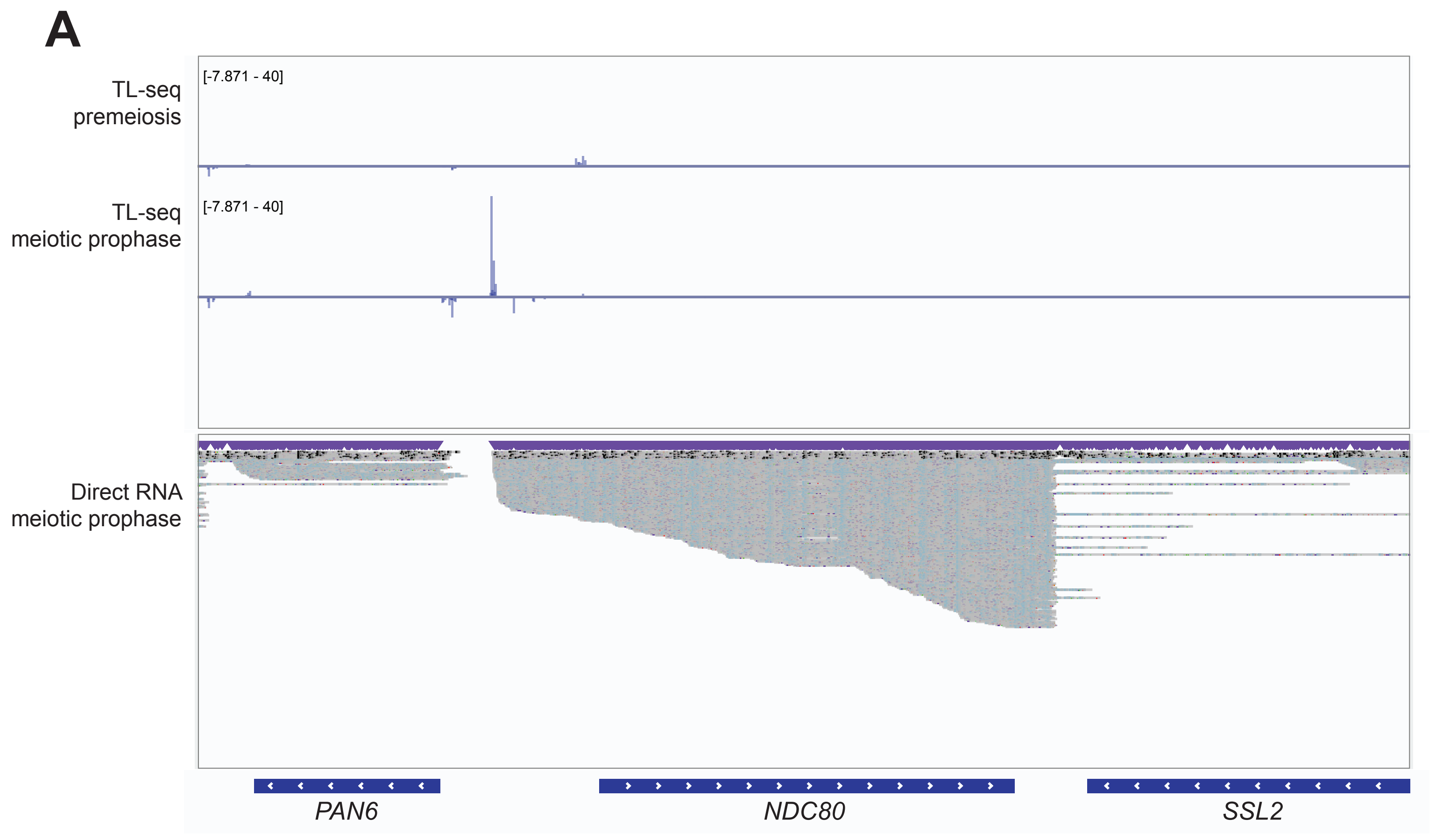

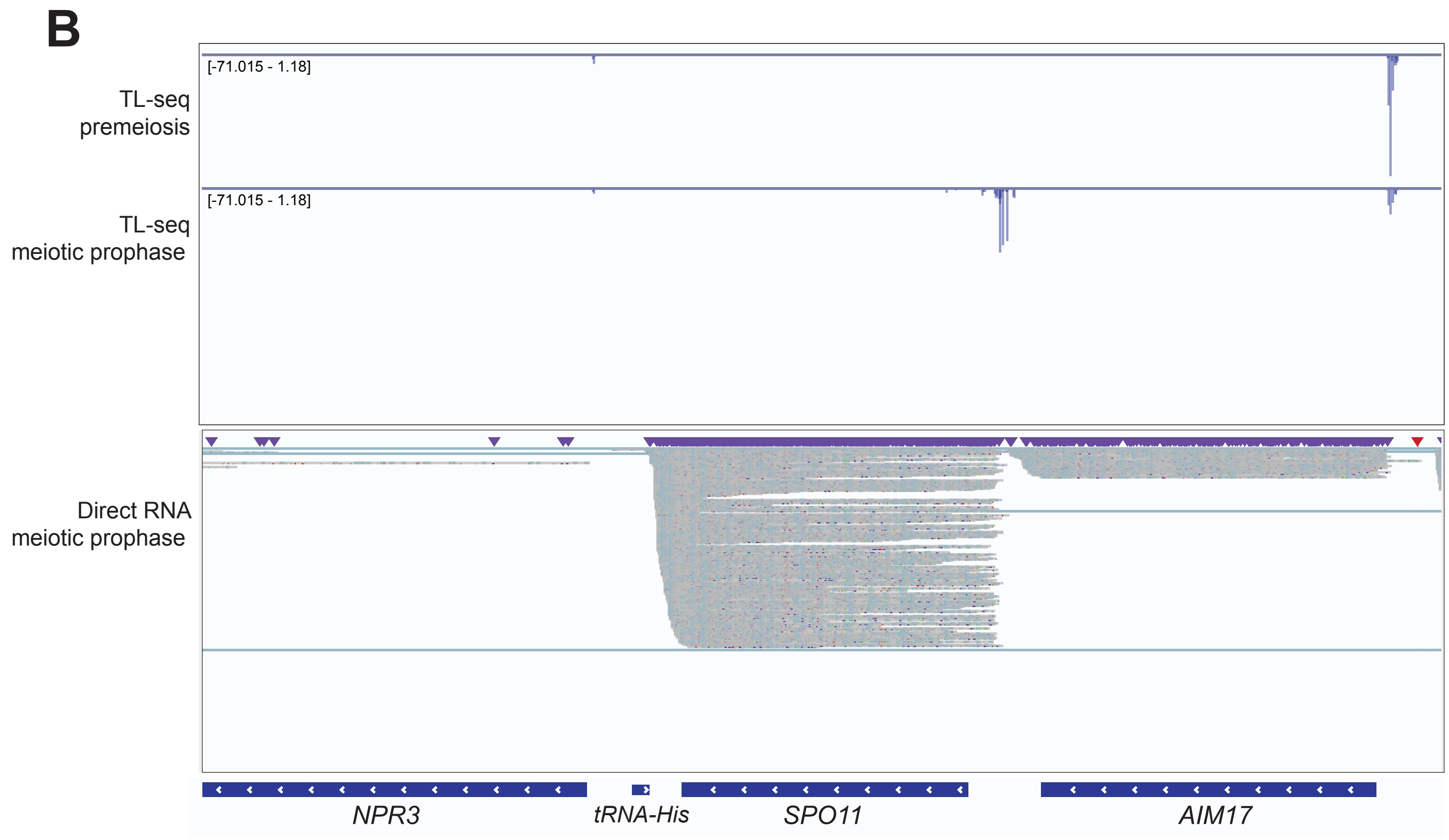

Figure S1, Tresenrider et al.

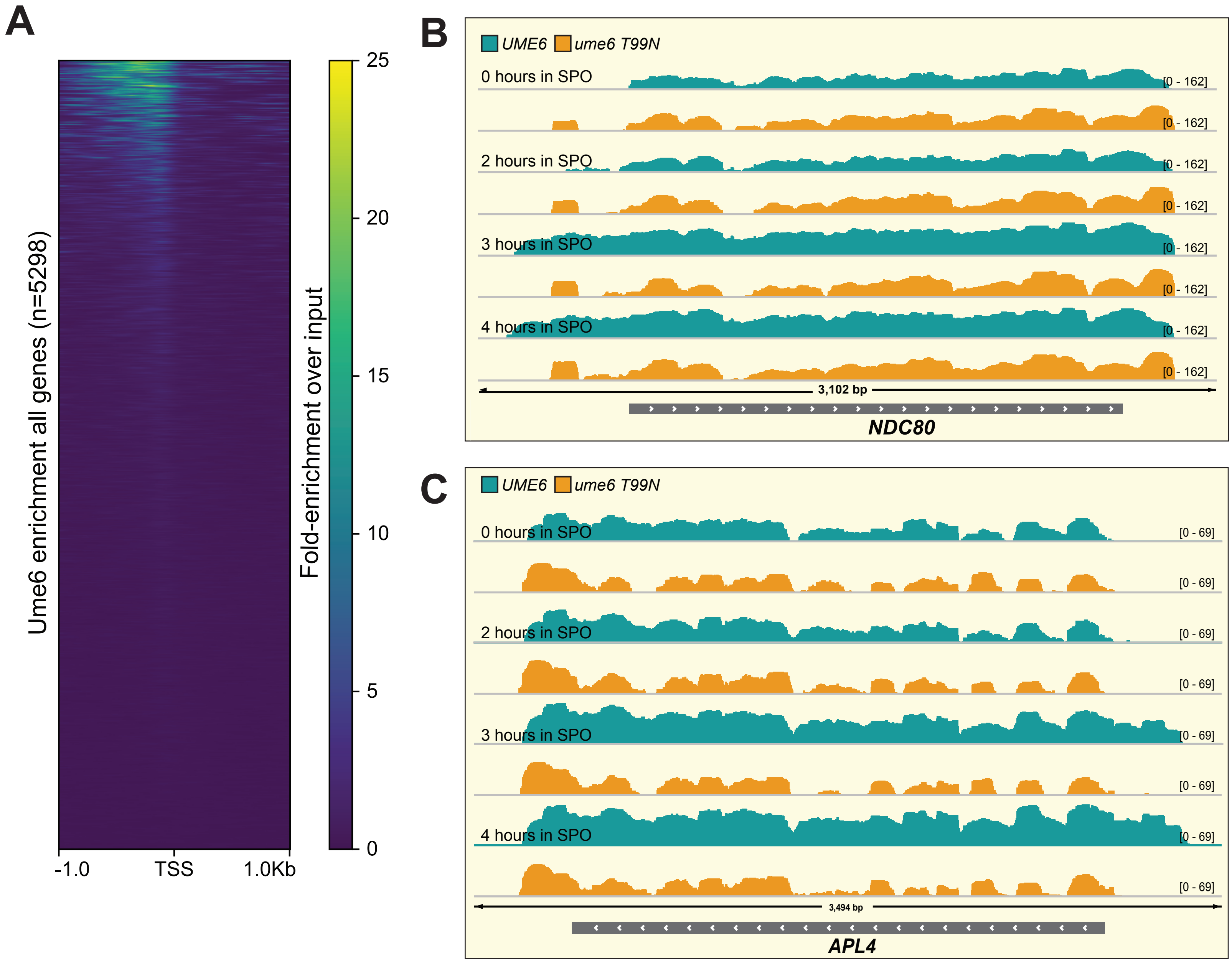

Figure S2, Tresenrider et al.

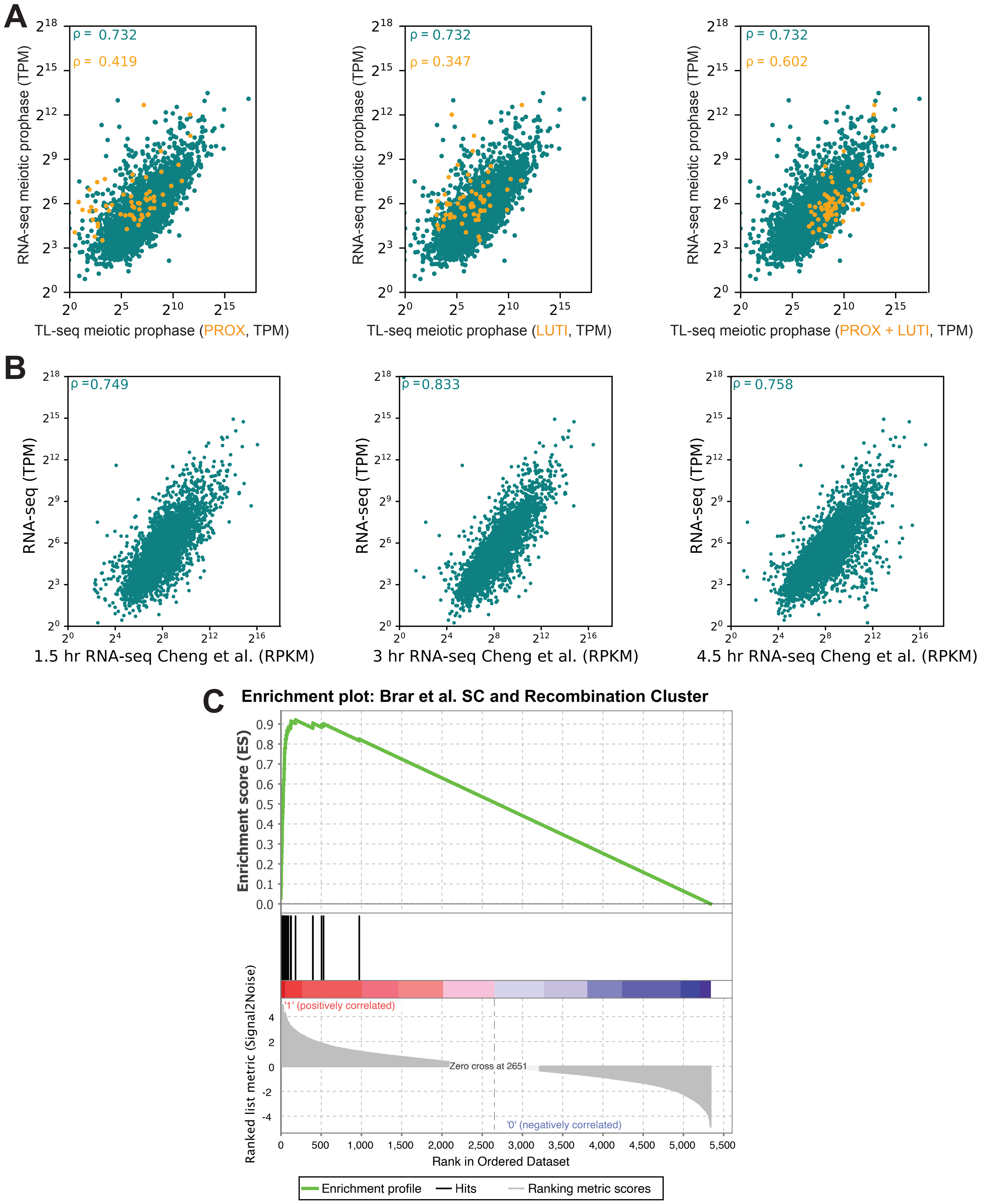

Figure S3, Tresenrider et al.

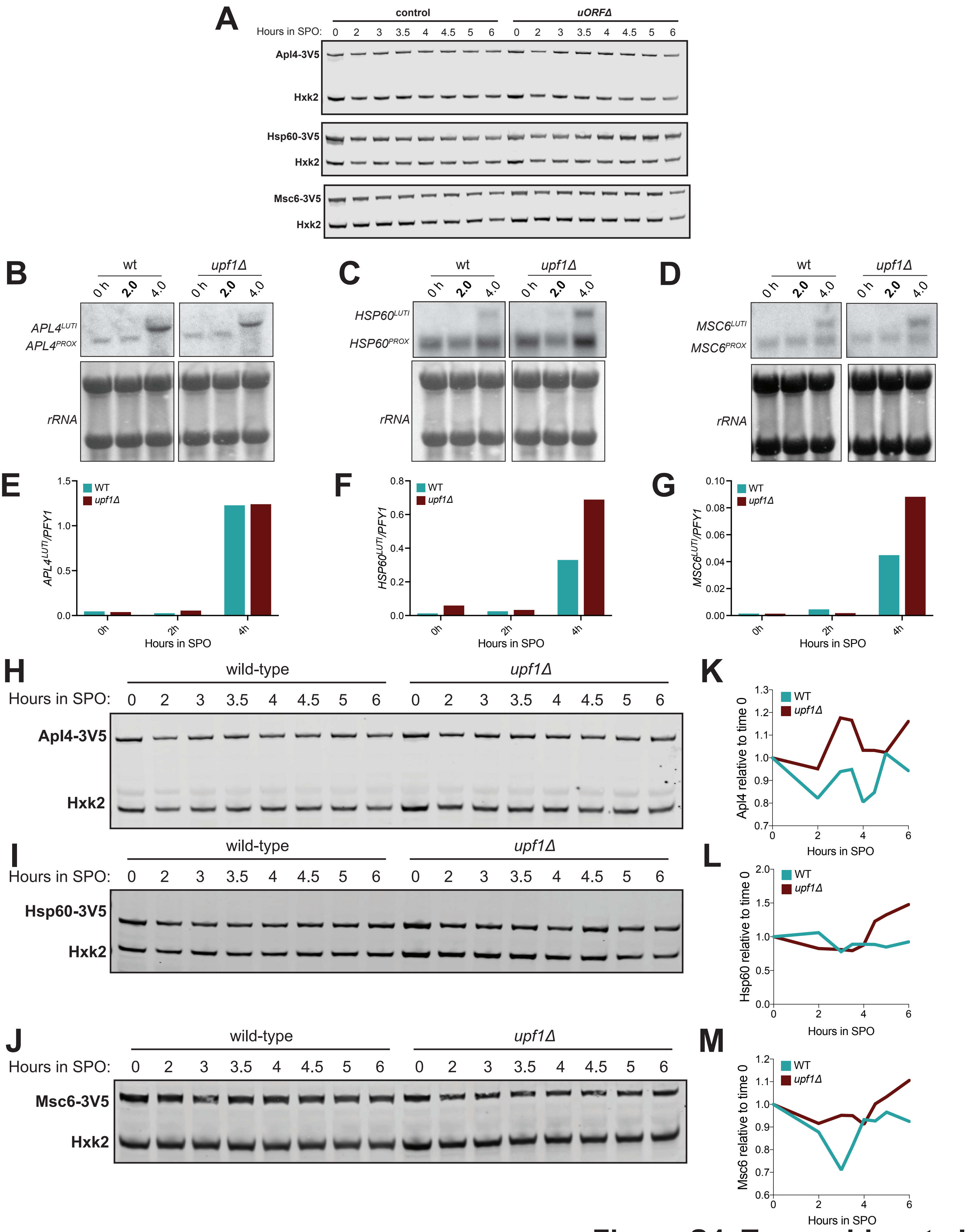

Figure S4, Tresenrider et al.

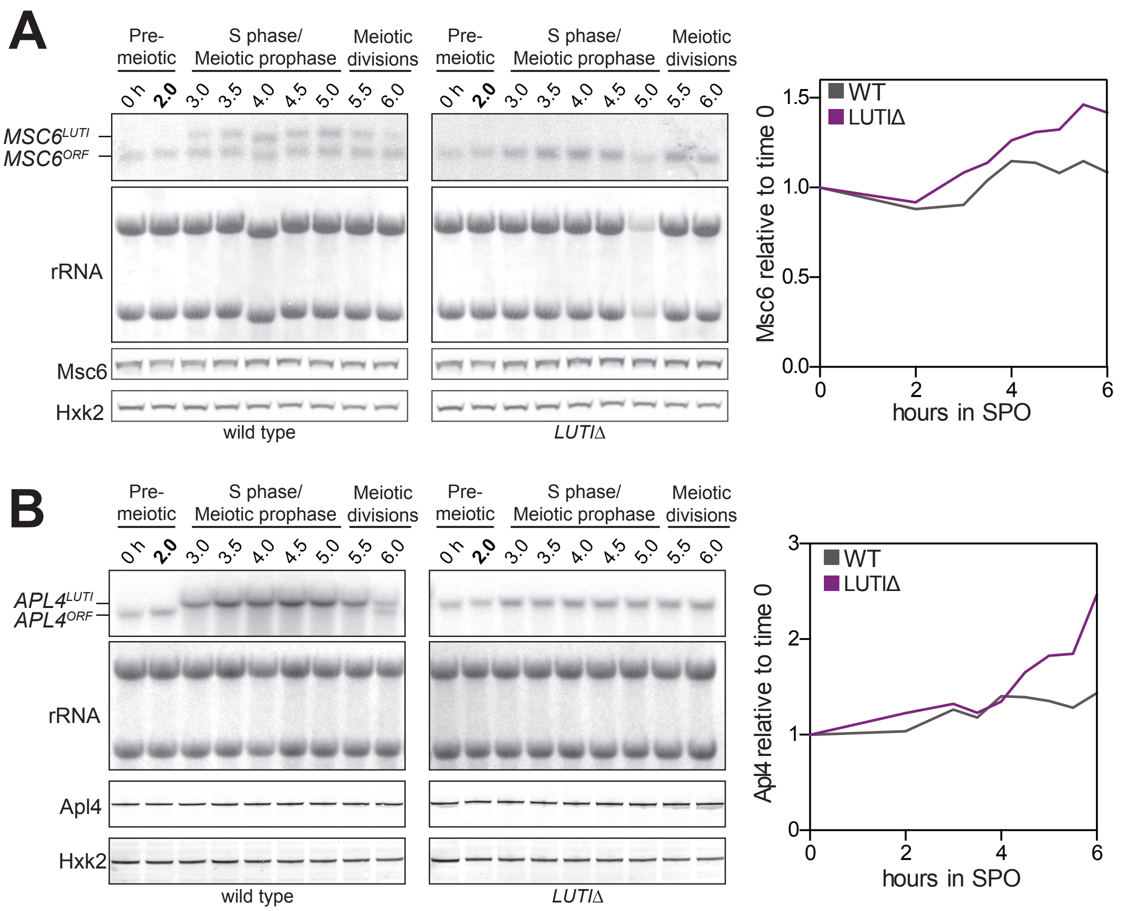

Figure S5, Tresenrider et al.

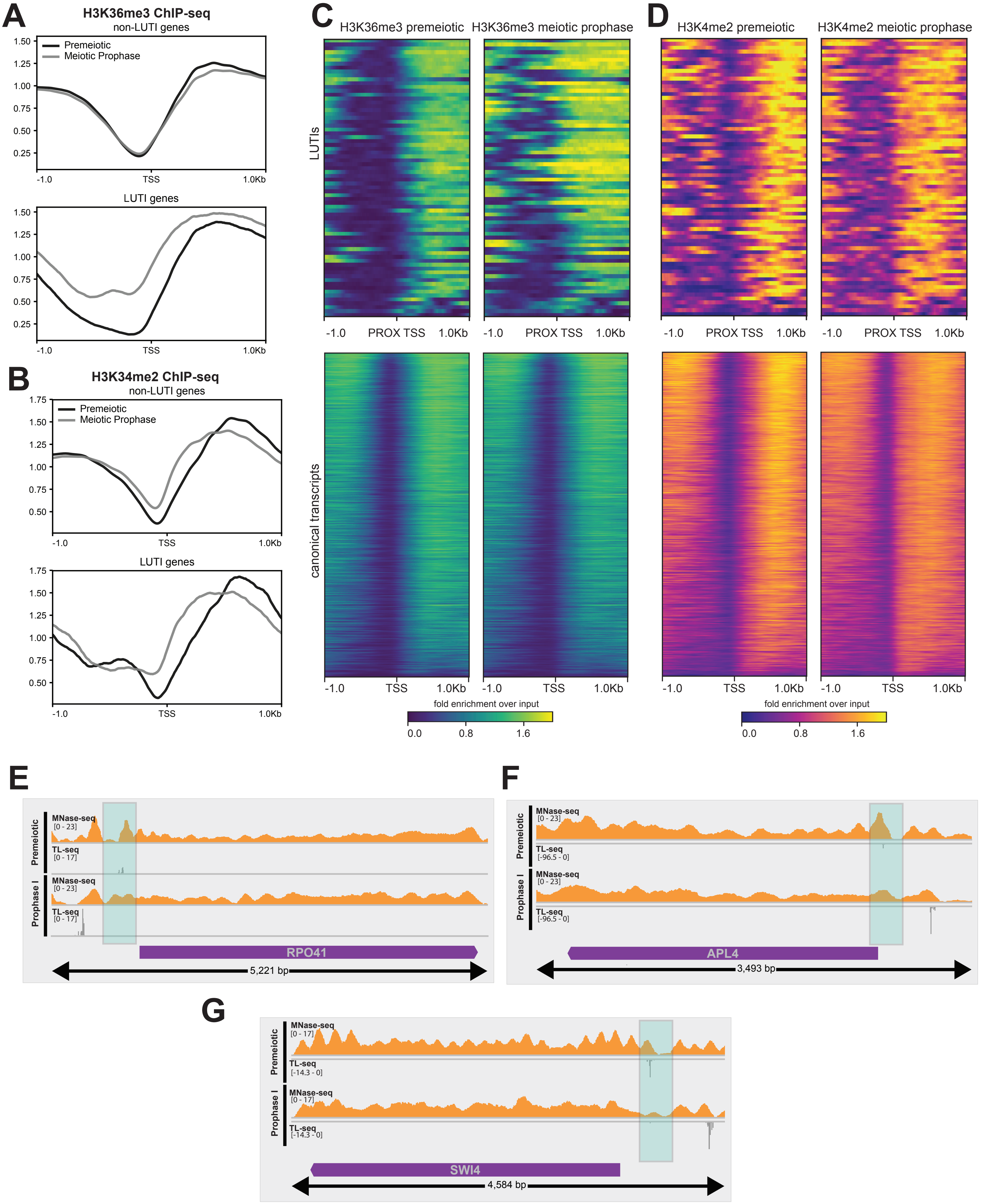

Figure S6, Tresenrider et al.

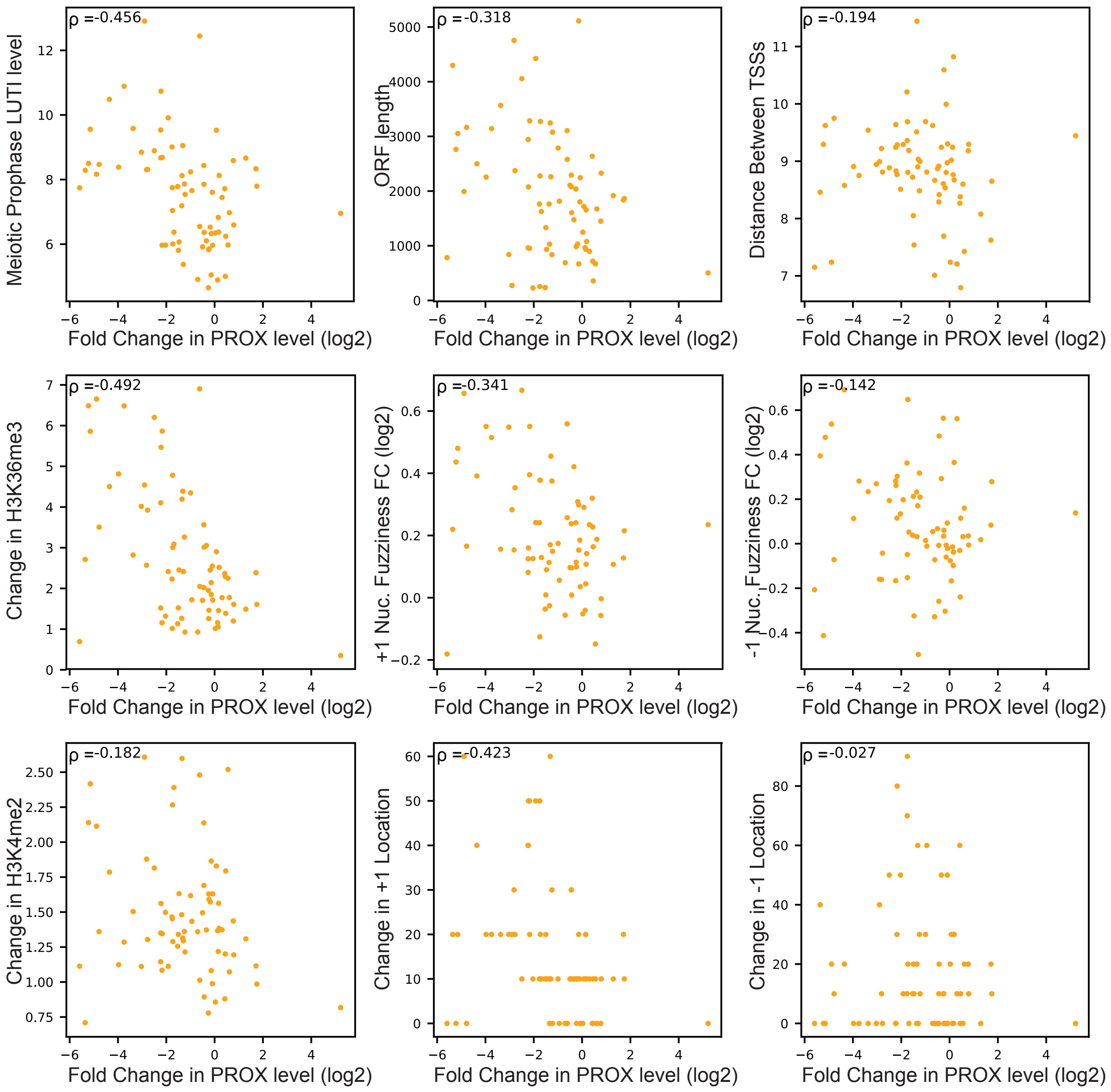

Figure S7, Tresenrider et al.

Table S5. LUTI candidates with fewer than 4 uORFs

Gene	uORF number	TE fold-change
PLM2	0	4.894
YCL057C-A	0	3.411
ELO1	1	1.078
COX16	1	0.995
ULP2	1	0.057
MNE1	1	0.005
ITR2	2	1.906
YNL305C	2	0.193
VPS30	3	0.236
APL4	3	0.193

Table S7. Primers used in this study.

Primer Name	Oligonucleotide sequence from 5' to 3'
3V5_probe_F	CTAGTGGATCCAGGTAAACCTAT
3V5_probe_R	TAATACGACTCACTATAGGCCAGTCCTAATAGAGGATTAGG
ACT1_F	GTACCACCATGTTCCCAGGTATT
ACT1_R	AGATGGACCACTTTCGTCGT
PFY1_F	ACGGTAGACATGATGCTGAGG
PFY1_R	ACGGTTGGTGGATAATGAGC
HSP60_F	TGGTTGCGTCTTCATGCAC
UBI_R	GGTCAAAGTCTTGACGAAAATCTG
APL4_qPCR_F	GCTACAGGAAGGTGGTGAAAG
APL4_qPCR_R	CTCGCAACCTCTCTACCTTC
HSP60_qPCR_F	CGCCAAGACTAGTTGTGCAA
HSP60_qPCR_R	CAGATACAGATGGGCGGCTA
MSC6_qPCR_F	AGGTTGCTCGTCGACTAAGT
MSC6_qPCR_R	GTGCAGAGCCAACTTAAGCA
