# Supplementary material for: Integrated genomic analysis reveals key features of long undecoded transcript isoform-based gene repression
Source: Mol Cell. 2021 May 20;81(10):2231–2245.e11. doi: 10.1016/j.molcel.2021.03.013 (PMC8153250; doi:10.1016/j.molcel.2021.03.013)
Supplement: Document S2. Article plus Supplemental information [file mmc7.pdf]

Molecular Cell

Article

Integrated genomic analysis reveals key features of long undecoded transcript isoform-based gene repression

Graphical abstract

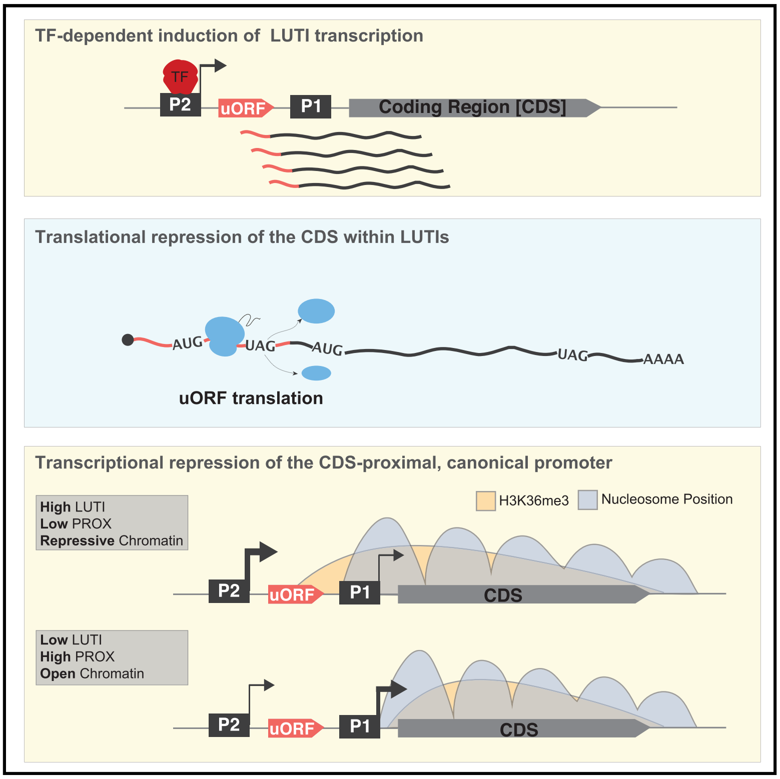

Highlights

- Long-read RNA-seq reveals 5′-extended mRNAs as candidate LUTIs in yeast meiosis
- Majority of the LUTIs are regulated by a cell-fate-instructive transcription factor
- LUTI-based translational repression depends on uORFs
- Chromatin changes and LUTI expression predict LUTI-based transcriptional repression

Authors

Amy Tresenrider, Kaitlin Morse, Victoria Jorgensen, Minghao Chia, Hanna Liao, Folkert Jacobus van Werven, Elçin Ünal

Correspondence

elcin@berkeley.edu

In brief

Tresenrider et al. conducted a genome-wide study to dissect an unconventional gene regulatory mechanism whereby transcription factors could function as repressors by inducing non-canonical mRNAs called LUTIs. Their findings reveal that LUTI-based gene repression depends on a combination of factors, including translational repression, chromatin state changes, and LUTI expression.

Tresenrider et al., 2021, Molecular Cell 81, 2231–2245
May 20, 2021 © 2021 The Author(s). Published by Elsevier Inc.
https://doi.org/10.1016/j.molcel.2021.03.013

Molecular Cell

CellPress
OPEN ACCESS

Article

Integrated genomic analysis reveals key features of long undecoded transcript isoform-based gene repression

Amy Tresenrider,[1] Kaitlin Morse,[1] Victoria Jorgensen,[1] Minghao Chia,[2,3] Hanna Liao,[1] Folkert Jacobus van Werven,[2] and Elçin Ünal[1,4,*]

[1]Department of Molecular and Cell Biology, Barker Hall, University of California, Berkeley, Berkeley, CA 94720, USA
[2]The Francis Crick Institute, 1 Midland Road, NW1 1AT London, UK
[3]Genome Institute of Singapore, 60 Biopolis Street, Genome, #02-01, Singapore 138672, Singapore
[4]Lead contact
*Correspondence: elcin@berkeley.edu
https://doi.org/10.1016/j.molcel.2021.03.013

SUMMARY

Long undecoded transcript isoforms (LUTIs) represent a class of non-canonical mRNAs that downregulate gene expression through the combined act of transcriptional and translational repression. While single gene studies revealed important aspects of LUTI-based repression, how these features affect gene regulation on a global scale is unknown. Using transcript leader and direct RNA sequencing, here, we identify 74 LUTI candidates that are specifically induced in meiotic prophase. Translational repression of these candidates appears to be ubiquitous and is dependent on upstream open reading frames. However, LUTI-based transcriptional repression is variable. In only 50% of the cases, LUTI transcription causes downregulation of the protein-coding transcript isoform. Higher LUTI expression, enrichment of histone 3 lysine 36 trimethylation, and changes in nucleosome position are the strongest predictors of LUTI-based transcriptional repression. We conclude that LUTIs downregulate gene expression in a manner that integrates translational repression, chromatin state changes, and the magnitude of LUTI expression.

INTRODUCTION

Gene expression programs determine cellular identity, form, and function through extensive regulation. Key regulators of such programs include transcription factors (TFs), which initiate cascades of gene expression that ultimately drive changes in cellular state. While TFs are generally implicated in gene activation, less is known about how gene repression events can be coordinated with TF-dependent waves of gene activation.

New insights into TF-mediated gene repression came from the studies of an essential gene called *NDC80*. *NDC80* is tightly regulated during meiotic differentiation, the developmental program that generates gametes (Asakawa et al., 2005; Chen et al., 2017; Miller et al., 2012; Sun et al., 2011). Upon meiotic entry, a key TF complex, Ime1-Ume6, activates the distal *NDC80* promoter (P2) to induce a 5′-extended mRNA isoform (Figure 1A). This transcript cannot be translated into a functional protein because the upstream open reading frames (uORFs) in its 5′ leader inhibit the translation of the coding sequence (CDS). Instead of producing Ndc80 protein, the “long undecoded transcript isoform” or “LUTI” serves to inactivate the CDS-proximal *NDC80* promoter (P1). As a result, the protein-coding *NDC80* transcript is downregulated in meiotic prophase (Chen et al., 2017; Chia et al., 2017). Reliance of the LUTI-based repression of *NDC80* on Ime1-Ume6 couples gene activation and repression events to a common TF.

The LUTI-based mechanism is neither limited to *NDC80* nor restricted to yeast meiosis. Genome-wide analysis revealed that at least 380 LUTIs are expressed throughout yeast meiosis, whereby transcript levels inversely correlate with protein expression (Cheng et al., 2018). During the endoplasmic reticulum unfolded protein response, Hac1 TF induces at least 15 LUTIs, most of which are required for downregulating genes involved in cellular respiration (Van Dalfsen et al., 2018). Finally, the *MDM2* oncogene is regulated by a LUTI-based mechanism in human cells (Hollerer et al., 2019). The prevalence of LUTIs in yeast and their discovery in humans, along with the pervasive use of alternative transcription start sites (TSSs) and the frequency of uORFs all point to the conservation of LUTI-based gene repression across vast evolutionary time (Batut et al., 2013; Calvo et al., 2009; Chew et al., 2016; Johnstone et al., 2016; Kimura et al., 2006).

Many questions remain to be answered about LUTI-based gene repression. First, why do only some 5′-extended transcripts appear to repress gene expression while others do not? Is the difference due to lack of translational repression,

This is an open access article under the CC BY license (http://creativecommons.org/licenses/by/4.0/).

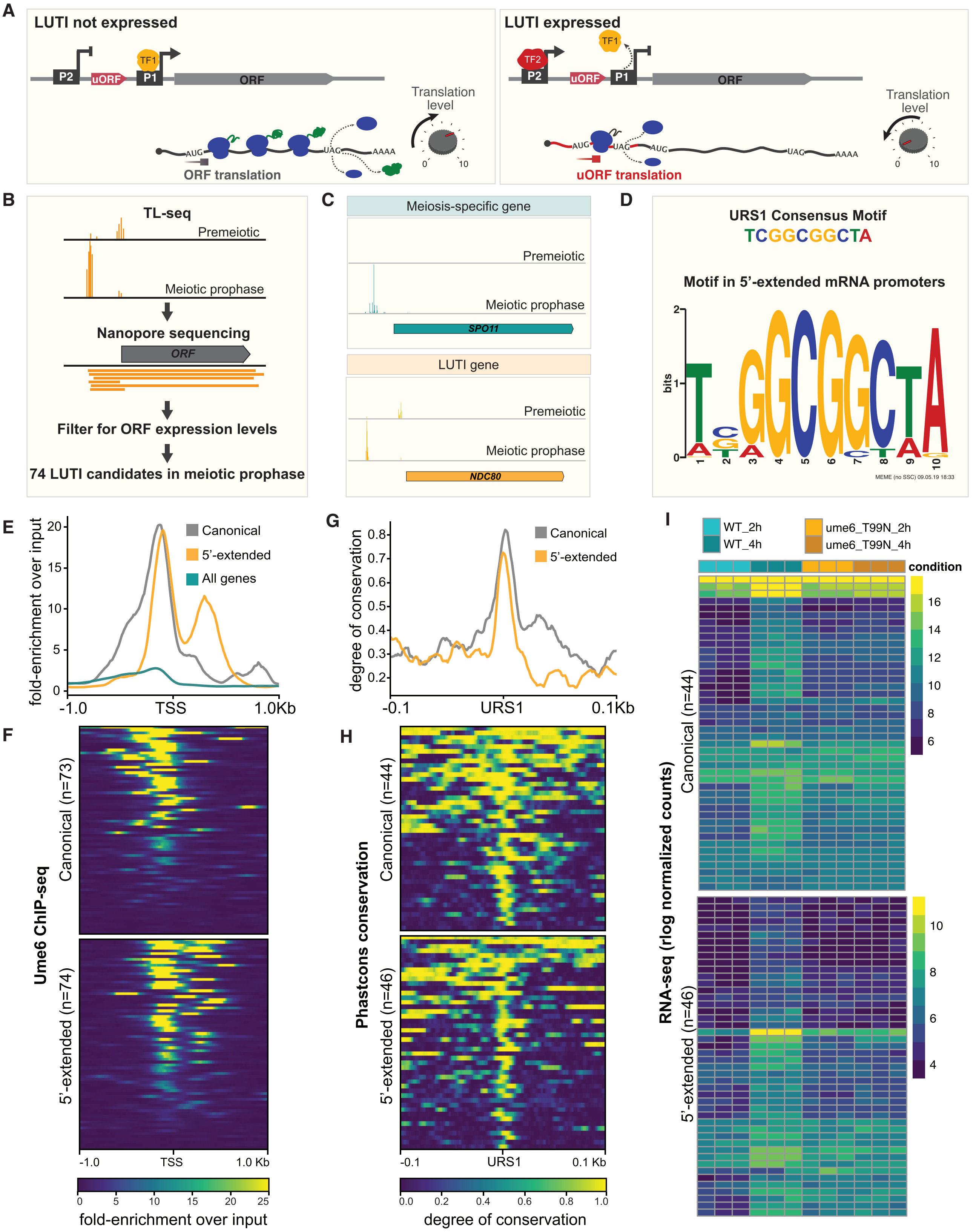

(legend on next page)

transcriptional repression, or both? Second, when gene repression does occur, does it involve a common mechanism? Lastly, what are the key features of the LUTI-based mechanism on a global scale?

In this study, we used transcript leader sequencing (TL-seq) in conjunction with RNA sequencing on nanopore arrays (direct RNA sequencing [RNA-seq]) to identify seventy-four 5′-extended transcript isoforms in meiotic prophase. A total of 80% of these transcripts are directly regulated by a meiotic TF with consensus-binding motifs conserved across the budding yeast *sensu stricto* genus. Importantly, uORFs are present in 97% of the 5′-extended transcripts and almost all of them appear to be bona fide LUTIs based on uORF-dependent translational repression. Transcriptionally, the outlook is more complex: LUTI transcription leads to the downregulation of the CDS-proximal transcript in 50% of cases. Chromatin modifications, specifically histone 3 lysine 36 trimethylation (H3K36me3), and changes in nucleosome position are among the strongest predictors of LUTI-based repression. Finally, higher LUTI expression leads to a greater repression of the CDS-proximal transcripts. We conclude that translational repression, chromatin state changes, and degree of LUTI expression act in a combinatorial manner to determine the extent of LUTI-based repression. Our findings provide a roadmap to uncover similar types of gene regulation in other biological contexts.

RESULTS

Transcript leader and nanopore sequencing identifies 74 potential LUTIs in meiotic prophase

To determine the prominent features of LUTI-based repression, we sought to identify meiotic mRNAs with 5′ extensions (Figure 1B). Using a previously published early meiotic cell synchronization system, we aimed to uncover all 5′-extended mRNAs that are expressed in meiotic prophase, but not in the premeiotic phase (Berchowitz et al., 2013). Identification of the 5′-extended isoforms relied on data from two complementary sequencing techniques. The first method, TL-seq, allowed for the identification of TSSs (Figures 1B and 1C; Arribere and Gilbert, 2013; Malabat et al., 2015). The second technique, nanopore sequencing, confirmed the instances in which TSSs identified by TL-seq produced transcripts that elongate across an entire CDS (Figure S1; Garalde et al., 2018). To distinguish LUTIs from the canonical meiotic mRNAs, we restricted our calls to loci in which the TSS identified by TL-seq was upstream of a second, CDS-proximal TSS (Figures 1B and 1C). These analyses revealed 74 potential LUTIs, 28 antisense transcripts, 65 intergenic transcripts, and 74 intragenic transcripts in meiotic prophase (Tables S1 and S2).

The vast majority of putative LUTIs are regulated by the same meiotic TF

We next looked for common regulatory features near candidate LUTI promoters and found a single significant hit matching the URS1 consensus motif (Figure 1D; Table S2; combined match $p < 0.05$) (Sumrada and Cooper, 1987). In mitosis, this motif is bound by Ume6, which functions as a transcriptional repressor (Park et al., 1992). However, Ume6 becomes a transcriptional co-activator upon interaction with the early meiotic TF Ime1, culminating in the expression of early meiotic genes (Bowdish et al., 1995).

To investigate how many URS1 sites are bound by Ume6, we performed chromatin immunoprecipitation followed by deep sequencing (ChIP-seq). Ume6 was enriched at 61 of the 74 candidate LUTI promoters ($q < 0.001$, fold enrichment > 4). The extent of enrichment was similar to a set of previously identified Ume6 targets (canonical, Figures 1E–1H; Williams et al., 2002). Both groups were more enriched with Ume6 than genes not in these lists (Figures 1E, 1F, and S2A; Table S2).

We further examined URS1 motif conservation in putative LUTI promoters. Using an alignment of 5 yeast species in the *sensu stricto* genus, conservation of the region around the URS1 motif was calculated for all of the sites that had both a URS1 motif and were bound by Ume6. For the previously defined canonical Ume6 targets and the regions around the newly identified 5′ extensions, conservation sharply increased around the URS1 motif (Figures 1G and 1H), providing strong evidence that 33 of the identified 5′-extended transcript isoforms have strongly conserved URS1 binding sites (Table S2).

As a final test, we performed RNA-seq in cells containing a mutant allele of *UME6* (T99N). The T99N mutation inhibits the interaction between Ume6 and Ime1, preventing meiosis-specific gene activation without compromising Ume6 function during mitosis (Bowdish et al., 1995). In these cells, only 2.7% of

Figure 1. LUTI discovery and analysis of TF-based regulation of LUTIs

(A) A schematic of LUTI-based gene regulation. The terms ORF and CDS are used interchangeably throughout the text and figures.

(B) An overview of the discovery pipeline.

(C) Genome browser views of TL-seq for the meiosis-specific gene *SPO11* and the *NDC80* locus, which contains a LUTI.

(D) The URS1 motif found in LUTI promoters and the consensus-binding motif observed 300 bp ± distal TSSs.

(E and F) Ume6 ChIP-seq from (*UB3301*) grown in BYTA to saturation. One of 3 replicates is shown.

(E) Metagene analysis of Ume6 fold enrichment over input in the promoters of all genes compared to the promoters of previously identified Ume6 targets (Williams et al., 2002) and to the promoters of the 5′-extended transcripts identified in this study.

(F) Heatmap of Ume6 fold enrichment over input in the promoters of previously identified Ume6 targets and in the promoters of the 5′-extended transcripts.

(G and H) For genes with both Ume6 enrichment and a URS1 motif, the degree of conservation across the 100 bp ± the URS1 motif center was determined by phastCons within the *sensu stricto* genus.

(G) Metagene analysis for degree of conservation in the promoters of previously identified Ume6 targets compared to the promoters of the 5′-extended transcript.

(H) Heatmap of degree of conservation in the promoters of previously identified Ume6 targets and in the promoters of the 5′-extended transcripts.

(I) RNA-seq from strains expressing either WT Ume6 (*UB20649*) or Ume6 with a T99N mutation (*UB22629*). Cells harboring *pCUP1-IME1*/*pCUP1-IME4* were collected after 2 h (premeiotic) or 4 h (meiotic prophase) in sporulation medium (SPO), which corresponds to 2 h after induction of meiosis by 50 μM $CuSO_4$. This induction method and timing was used for all subsequent experiments.

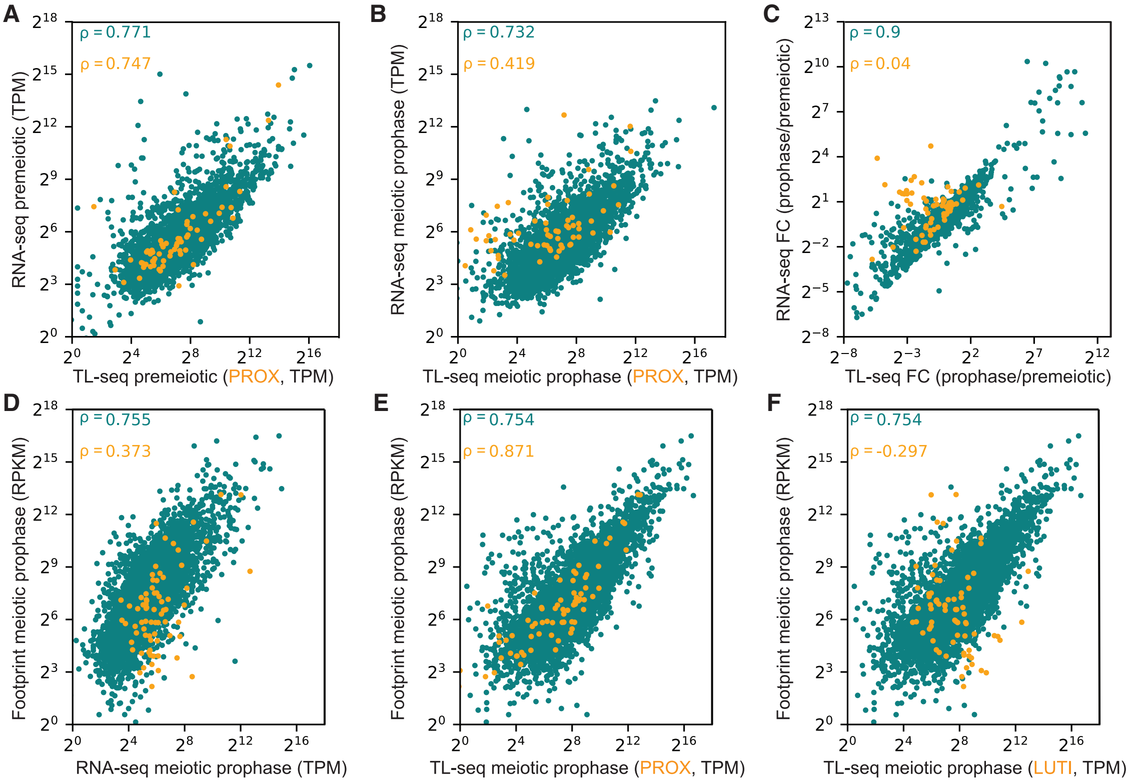

Figure 2. Relationship between transcript isoform diversity and translational regulation of 5′-extended isoforms

(A–F) Scatterplots comparing genes with (orange) and without (teal) 5′ extensions. Spearman's rank correlation coefficients (ρ) displayed.

(A and B) Scatterplot of RNA-seq and TL-seq premeiosis (A) and during meiotic prophase (B). For genes with 5′ extensions, only the PROX transcript is quantified by TL-seq (n = 2).

(C) The fold change (FC) by which gene expression changes as the cells enter meiotic prophase from premeiotic stage.

(D–F) Scatterplot of translation as measured by ribosome footprints versus (D) RNA-seq (n = 2) and (E and F) TL-seq (n = 3). In (E), only the PROX transcript is quantified for genes with 5′ extensions, and in (F) only the LUTI transcript is quantified for genes with 5′ extensions. The ribosome profiling data are from the 3-h time point in Cheng et al. (2018).

the loci with 5′-extended transcripts had increased gene expression after the induction of *IME1* (Figures 1I, S2B, and S2C; Table S3). The percentage was reduced to 2.2% for the 46 loci in which a URS1 motif is present and Ume6 binding is detected by ChIP-seq. In combination, this evidence suggests that Ime1-Ume6 directly regulates the vast majority of 5′-extended transcripts identified in this study (Table S3).

Transcript isoform diversity and the translational regulation of 5′-extended isoforms

LUTIs were originally identified based on an inverse correlation between transcript and protein levels (Chen et al., 2017; Cheng et al., 2018). Of the 380 meiotic LUTIs in Cheng et al. (2018), 32 (8.4%) of them were found to be expressed during the meiotic prophase time point collected in this study. The remaining 42 of our 74 candidate LUTIs were previously unidentified. While 10 of the false negatives were missed due to the lack of a quantifiable protein measurement, the other 32 were likely overlooked due to differences in the method of identification (Cheng et al., 2018). Our pipeline detects all 5′-extended transcripts, regardless of their correlation with protein abundance. Such newly identified LUTI candidates are potentially very interesting because they may reveal biological insights into how frequently candidate LUTIs repress gene expression and under what circumstances this repression occurs.

It is difficult to answer these questions using traditional RNA-seq, because the transcript produced from the CDS-proximal promoter (PROX isoform) does not have any unique identifying sequence compared to the transcript produced from the CDS-distal promoter. By sequencing the most 5′ end of a transcript with TL-seq, this was no longer a limitation. We performed TL-seq and RNA-seq in parallel on samples collected during the premeiotic phase and meiotic prophase. RNA-seq and TL-seq measurements (in which the closest TSS to CDS was quantified) correlated well for most genes, demonstrating that TL-seq can quantitatively estimate transcript abundances (Spearman's rank correlation coefficient, $\rho = 0.771$ [premeiotic], $\rho = 0.732$ [meiotic prophase]; Figures 2A and 2B). However, for genes with 5′-extended transcripts, this correlation was poor in meiotic prophase for both the PROX ($\rho = 0.419$) and extended ($\rho = 0.347$) transcripts; it improved when both transcripts were considered ($\rho = 0.602$) (Figures 2B and S3A). When the fold change (FC) of expression between meiotic prophase and premeiotic stages was taken into account, there was no

CellPress
OPEN ACCESS

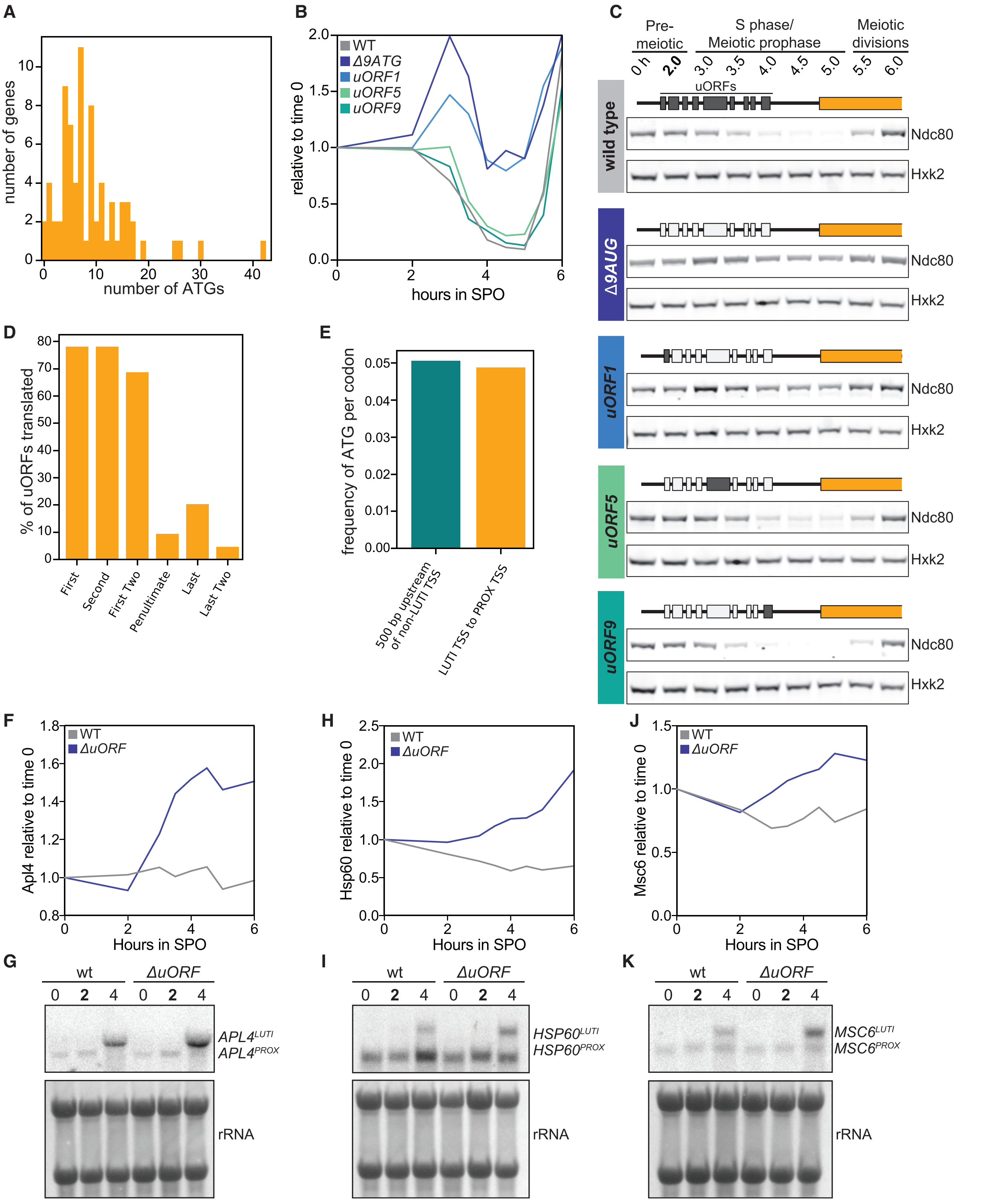

(legend on next page)

correlation between TL-seq and RNA-seq for genes with 5′-extended transcripts (ρ = 0.04; Figure 2C). This suggests that TL-seq is a more robust method than RNA-seq to specifically quantify the 5′-extended and PROX isoforms.

With the ability to distinguish between the transcript isoforms, we set out to compare the correlation between CDS translation and transcript isoform abundance using a published dataset (Cheng et al., 2018). The meiotic prophase RNA-seq dataset from our study (4-h) best matched the 3-h time point from Cheng et al. (2018), which used asynchronous meiotic cultures (ρ = 0.833; Figure S3B). This 3-h time point represents the peak period when recombination and synaptonemal complex formation occurs (Cheng et al., 2018; Brar et al., 2012). Gene set enrichment analysis (GSEA) from our RNA-seq dataset also revealed that recombination-related gene sets were highly enriched in the corresponding 4-h time point (Table S4; Mootha et al., 2003; Subramanian et al., 2005). The most enriched gene set was that of genes previously reported as highly induced in the 3-h time point from asynchronous meiotic cultures (Figure S3C; Table S4). These metrics gave us confidence in comparing datasets from the two studies.

For genes without 5′ extensions, the distribution between RNA-seq and ribosome footprints looked very similar to the distribution between TL-seq and footprints (ρ = 0.755 versus ρ = 0.754; Figures 2D–2F). However, for genes with 5′ extensions, there were fewer footprints per unit mRNA relative to the rest of the transcriptome, indicating poor translation (ρ = 0.373; Figure 2D). Remarkably, the PROX transcripts from TL-seq were highly proportional to the translation measurements (ρ = 0.871; Figure 2E), while the 5′-extended transcripts showed a negative correlation (ρ = −0.297; Figure 2F). This analysis demonstrates that transcript isoform-specific measurements are more accurate in assessing translational status than RNA-seq and provides additional evidence that the 5′-extended transcripts do not productively translate the CDSs contained within them.

Prevalence of uORFs in meiotic LUTIs

We next investigated how the LUTIs were translationally impaired. While 5′ leader-mediated translational repression can occur through various means (Hinnebusch et al., 2016), the abundance of uORFs in meiosis (Brar et al., 2012), as well as the uORF-dependent translational repression of *NDC80^{LUTI}* (Chen et al., 2017), led us to hypothesize that uORFs would play a dominant role in dampening CDS translation from LUTIs.

The number of ATG codons in the region between the distal TSS and the PROX TSS was used to determine the number of uORFs in candidate LUTIs. This analysis revealed that 72 of 74 candidate LUTIs had at least 1 uORF, and 64 had between 4 and 17 (Figure 3A). The two without a uORF (*PLM2* and *YCL057C-A*) displayed an increase in TE (translational efficiency = ribosome footprints reads per kilobase of transcript, per million mapped reads [RPKM]/transcript abundance RPKM), indicating that they should not be categorized as LUTIs (Table S5).

When an extended transcript contained a single uORF, the effect on translational efficiency was variable. The TE changed very little for 2 genes (*ELO1* and *COX16*), possibly because the LUTI was the minor isoform. The LUTI was the major isoform in the other 2 cases, and the TE decreased dramatically (Table S5), suggesting that a single uORF was sufficient to inhibit translation initiation at downstream AUGs. Based on these analyses, all of the 5′-extended transcripts containing at least a single ATG uORF will be referred to as LUTIs going forward.

We directly tested the ability of a single uORF to repress the translation of the downstream CDS at the *NDC80* locus. *NDC80^{LUTI}* normally contains 9 uORFs. When all 9 ATGs were mutated to ATCs (*Δ9AUG*), Ndc80 protein was translated from the LUTI (Figures 3B and 3C; Chen et al., 2017). Strains were constructed in which the ATG of either the first, fifth, or ninth uORF was the sole ATG-initiated uORF in the 5′ leader. Having the first uORF alone resulted in similar Ndc80 protein levels to what was observed in the *Δ9AUG* strain; however, LUTIs carrying only the fifth or the ninth uORF repressed Ndc80 translation just as efficiently as the wild-type *NDC80^{LUTI}* (Figures 3B and 3C).

We next set out to establish another metric to determine whether LUTIs are translationally repressed. As the uORF number in a 5′ leader increases, the likelihood of repression at the downstream CDS also increases (Calvo et al., 2009; Chew et al., 2016; Johnstone et al., 2016). We predicted that if translational repression occurs, then there would be more translation over the most 5′ uORFs and less translation over the uORFs closest to the CDS. Because uORFs are short and frequently overlapping, it can be difficult to accurately quantify their translation. Instead, we determined a threshold of at least 4 footprint counts within the first 6 codons of a uORF to call it as translated. With this metric, almost 80% of the first 2 uORFs in transcripts with at least 4 uORFs were classified as translated (Figure 3D), while <15% of the last 2 uORFs in those same transcripts were translated (Figure 3D). Thus, the ribosomes

Figure 3. uORF-based translational repression is prevalent in LUTIs

(A) A histogram of the number of ATGs found in the region between the proximal and the distal TSSs at loci with LUTI mRNAs.

(B and C) Cells with 3V5-tagged *NDC80* were induced to undergo meiosis. Strains: wild type (*UB6190*), *Δ9AUG* (all of the ATGs in the *NDC80^{LUTI}* leader mutated to ATC, *UB6183*), and strains in which only the first (*uORF1: UB10579*), fifth (*uORF5: UB10581*), or ninth (*uORF9: UB10583*) ATG was left intact. Immunoblots with α-V5 antibody (Ndc80-3V5). Hxk2 was used as a loading control. (n = 2). (B) Quantification of the blots. (C) Immunoblots from (B).

(D) The frequency of uORF translation in LUTIs containing at least 4 uORFs. The translation state of the 2 most 5′ and the 2 most 3′ AUG uORFs were assessed.

(E) The frequency of ATGs in the region between the LUTI TSS and the CDS-proximal TSS was compared to the region 500 bp upstream of TSS that do not express LUTIs in meiotic prophase.

(F–K) Cells were induced to enter meiosis after 2 h in SPO.

(F and G) *APL4-3V5* with either wild type (*UB18539*) or mutated uORFs (*UB26122*).

(H and I) *HSP60-3V5* with either wild type (*UB18336*) or mutated uORFs (*UB26123*).

(J and K) *MSC6-3V5* with either wild type (*UB18238*) or mutated uORFs (*UB26124*).

(F, H, and J) Immunoblots against the 3V5 epitope were quantified. One of 2 replicates.

(G, I, and K) RNA blots performed with a probe specific for 3V5 and its linker. rRNA bands detected by methylene blue.

CellPress
OPEN ACCESS

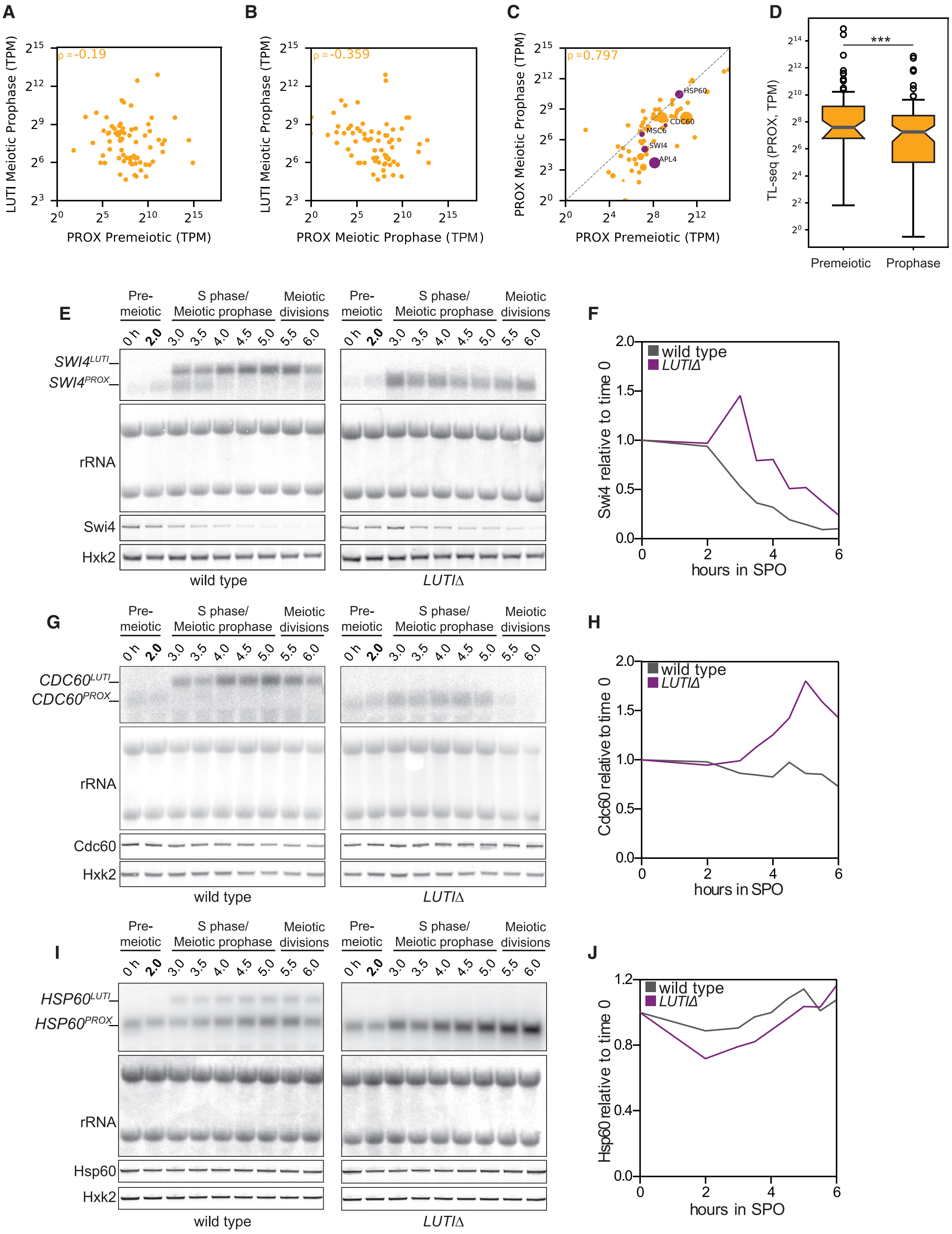

(legend on next page)

frequently are caught up before scanning across all uORFs. This is consistent with the observation that ATG frequency was not higher in the 5′ extensions compared to the 500 bp upstream of genes not expressing LUTIs in meiotic prophase (Figure 3E). If LUTIs do play important and functional roles in mediating meiotic gene expression, then the lack of uORF selection would indicate that the natural frequency of ATGs in intergenic regions is sufficient to result in the necessary degree of translational inhibition. We conclude that uORFs are found in abundance in the 5′ leaders of most LUTIs.

Using three LUTIs (*APL4*, *HSP60*, and *MSC6*), we further tested the requirement of uORFs in translational repression. The ATG of all uORFs in the 5′ leaders of these LUTIs were mutated to ATC (Δ*uORF*). Cells from wild-type and Δ*uORF* strains were synchronized in meiosis. Compared to cells with wild-type uORFs, the protein abundance was higher in the Δ*uORF* strains at the 4-h time point (Figures 3F, 3H, 3J, and S4A). This occurred even in the absence of any observed increase in the PROX transcript, suggesting that increased translation was occurring from the mutated LUTI transcript (Figures 3G, 3I, and 3K). Intriguingly, an increase in LUTI levels was evident in the Δ*uORF* mutants (Figures 3G, 3I, and 3K). However, this alone is not sufficient to alter protein abundance as observed in *upf1Δ* cells, which have higher LUTI levels due to deficiencies in nonsense-mediated mRNA decay, a degradation pathway for transcripts with premature translation-termination codons (Figures S4B–S4M). Our findings indicate that the presence of uORFs is a major driving factor behind the poor translational efficiency of LUTIs.

Variable transcriptional repression by LUTIs

To assess the degree of transcriptional repression, the abundances of LUTI and PROX transcripts were measured by TL-seq. LUTI levels did not correlate with PROX transcript abundance before meiotic entry ($\rho = -0.19$; Figure 4A); however, a significant negative relationship developed in meiotic prophase ($\rho = -0.359$, $p = 1.98 \times 10^{-3}$), associating LUTI expression with a decrease in PROX isoform level (Figure 4B). In addition, the PROX transcript abundance in meiotic prophase was less than in the premeiotic stage for a large number of genes (Figures 4C and 4D).

To parse out how causative the relationship was between LUTI and PROX expression, five genes were selected for in-depth analysis. *SWI4* and *APL4* had very strongly repressed PROX transcripts in meiotic prophase, *MSC6* and *HSP60* had PROX transcripts present at similar levels in both time points, and *CDC60* had an intermediate amount of PROX repression (Figure 4C). Transgenes carrying an epitope tag with either a normal LUTI promoter (wild type) or a deletion (Δ*LUTI*) were constructed. No deletions affected PROX expression compared to wild type in the premeiotic phase, but in all instances, the LUTI deletion led to an increase in meiotic PROX transcript (Figures 4E, 4G, 4I, S5A, and S5B). Increases in protein levels for Swi4 and Cdc60 (Figure 4F and 4H), but not for Apl4, Msc6 and Hsp60, were observed (Figures 4J, S5A, and S5B). Thus, even when LUTI-based repression is relieved and PROX transcript isoform abundance increases, additional layers of control can buffer the final protein output.

The role of chromatin in LUTI-based transcriptional repression

With the knowledge that LUTI expression can play a role in repressing transcription from downstream TSSs at some loci, but not others, we set out to understand what differentiates the two cases. In both yeast and human cells, LUTI expression leads to an increase in H3K36me3 over the proximal gene promoter (Chia et al., 2017; Hollerer et al., 2019). In yeast, *NDC80*^{LUTI} expression also results in H3K4me2 enrichment (Chia et al., 2017). Both marks are necessary for the LUTI-based repression of *NDC80* (Chia et al., 2017).

To determine the change in H3K36me3 and H3K4me2 patterns at LUTI-associated loci, we performed ChIP-seq. In premeiotic cells, the LUTI-containing genes displayed dis-enrichment of both modifications just upstream of the PROX TSS (Figures 5A and 5B, top panel, and Figures S6A–S6D). Interestingly, H3K36me3 was enriched in the meiotic prophase specifically over the promoters of those genes expressing LUTIs (Figure 5A, bottom panel). Strikingly, those genes whose PROX transcript is most repressed (FC < 0.25, n = 21) have the highest levels of H3K36me3 over their proximal TSS (Figure 5A; Table S6). In contrast, genes with LUTIs that do not experience a decrease in the PROX transcript (log2 FC > 0, n = 18) have only a minor increase in H3K36me3 levels (Figure 5A; Table S6). For H3K4me2, a moderate increase in the chromatin modification is observed over the PROX promoters of only those genes that are the most repressed in meiotic prophase (Figure 5B; Table S6). Thus, while increased H3K36me3 is a strong predictor for LUTI-based repression, H3K4me2 appears to be altered in a limited number of instances.

Figure 4. Transcriptional repression by LUTIs

(A) Scatterplot of LUTI abundance in cells from meiotic prophase versus PROX abundance in premeiotic cells (*UB14584*) (n = 3). Spearman's correlation coefficient (ρ) is shown.

(B) The same as (A), except that the PROX transcript abundance is from the meiotic prophase time point.

(C) Scatterplot of PROX abundance in meiotic prophase compared to premeiotic cells. The size of each point correlates to the LUTI abundance in meiotic prophase. Purple points indicate genes selected for in-depth analysis.

(D) Boxplot of PROX transcript abundance in premeiosis and meiotic prophase (Wilcoxon signed-rank test: $p = 1.52 \times 10^{-4}$). Default box and whisker plot settings were used. The notch is at the median and the box extends across the middle quartiles. Whiskers include all data not considered outliers.

(E–J) Cells were induced to enter meiosis after 2 h in SPO.

(E and F) *SWI4-3V5* with either wild type (*UB18175*) or *LUTIΔ* (*UB18176*).

(G and H) *CDC60-3V5* with either wild type (*UB18185*) or *LUTIΔ* (*UB18186*).

(I and J) *HSP60-3V5* with either wild type (*UB18336*) or *LUTIΔ* (*UB18188*).

(E, G, and I) RNA blots with a probe specific for 3V5 and its linker. rRNA (methylene blue stain) was used as a loading control. Immunoblots were performed with a α-V5 antibody. Hxk2 was used as a loading control. One of 2 replicates for *CDC60* and *SWI4*.

(F, H, and J) Quantification of immunoblots in (E), (G), and (I).

CellPress
OPEN ACCESS

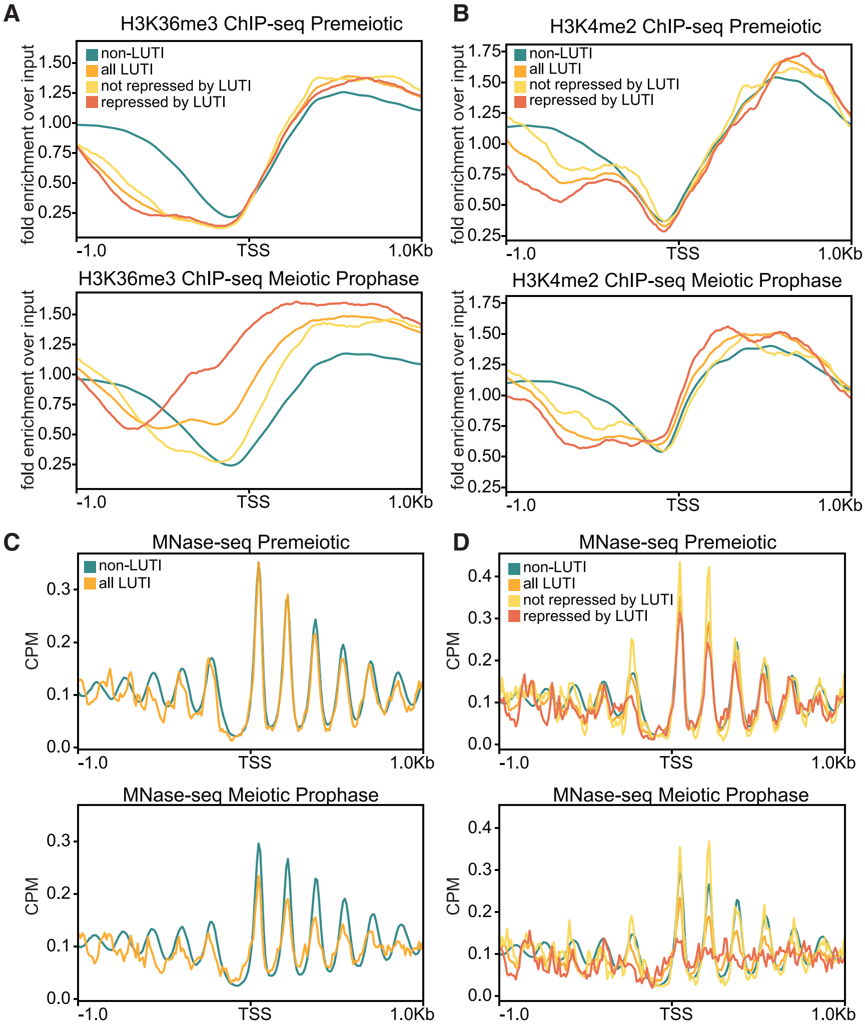

Figure 5. Chromatin modifications and nucleosome position in LUTI-based transcriptional repression

(A) Metagene analysis of H3K36me3 ChIP-seq. Repressed LUTI genes include the subset of genes with a FC in PROX abundance of <0.25 between the early meiotic and premeiotic time points (n = 21). The non-repressed LUTI genes are those with a FC in PROX abundance of >1 (n = 18). The plot is centered around the PROX TSS. One of 3 replicates.

(B) Same as (A), but for H3K4me2.

(C) Metagene analysis of MNase-seq. One of 3 replicates.

(D) Same as (C), but including the non-repressed and repressed gene groups from (A).

We next tracked genome-wide changes to nucleosome positions using micrococcal nuclease digestion with deep sequencing (MNase-seq) since the presence of nucleosomes can occlude the binding of TFs and machinery required for transcription initiation (Klemm et al., 2019; Venkatesh and Workman, 2015). In meiotic prophase, but not in the premeiotic stage, the nucleosome peaks near PROX promoters decreased while the signal in the valleys increased (Figure 5C; Chen et al., 2013). The effect was strongest for loci with the greatest degree of PROX transcript repression (Figure 5D). In that subset of genes, the nucleosome position was so disrupted that consensus nucleosome periodicity could not be identified (Figure 5D). Examining such a small subset of genes may make it hard to observe strong periodicity; however, in a similarly small subset of genes (n = 18), loci with non-repressive LUTIs, periodic nucleosome positioning was still present (Figure 5D). The complete lack of periodicity at loci with repressive LUTIs was most likely due to variability in the extent of repositioning at each locus (Figures S6E and S6F). We conclude that robust nucleosome repositioning occurs over the promoters of genes that are most subject to LUTI-based transcriptional repression.

The features defining LUTI-based transcriptional repression

We expected that other variables could play a role in LUTI-based transcriptional repression. Stemming from previous work showing that the distance between promoters with overlapping transcription affects the mechanism of transcriptional repression in cells undergoing carbon source shifts (Kim et al., 2016), we considered the importance of the distance between the PROX and the LUTI TSSs. The LUTI abundance and the length of the gene were also considered. To better assess how changes to the nucleosome over the PROX promoter may be associated with LUTI-based repression, the DANPOS2 toolkit was used to define changes to the +1 and −1 nucleosome positions and to assess "fuzziness," defined as the frequency with which the nucleosome differs from the most observed position (Chen et al., 2013). We found that an increase in H3K36me3 ($\rho = -0.492$, $p = 1.10 \times 10^{-5}$), high LUTI levels ($\rho = -0.456$, $p = 5.60 \times 10^{-5}$), +1 nucleosome peak moving toward the nucleosome-depleted region ($\rho = -0.423$, $p = 2.13 \times 10^{-4}$), and an increase in +1 nucleosome fuzziness ($\rho = -0.341$, $p = 3.41 \times 10^{-3}$) displayed significant negative correlation with the log2 FC of PROX transcript abundance (Figures 6A and S7). However, changes at the −1 nucleosome, H3K4me2, and the distance between the TSSs had no significant correlation (Figures 6A and S7). These analyses helped distinguish a set of important factors involved in LUTI-based transcriptional repression.

Promoter strengths dictate LUTI-based transcriptional repression

Surprisingly, we also found that the longer the coding sequence of a gene, the more likely it was to have an abundant

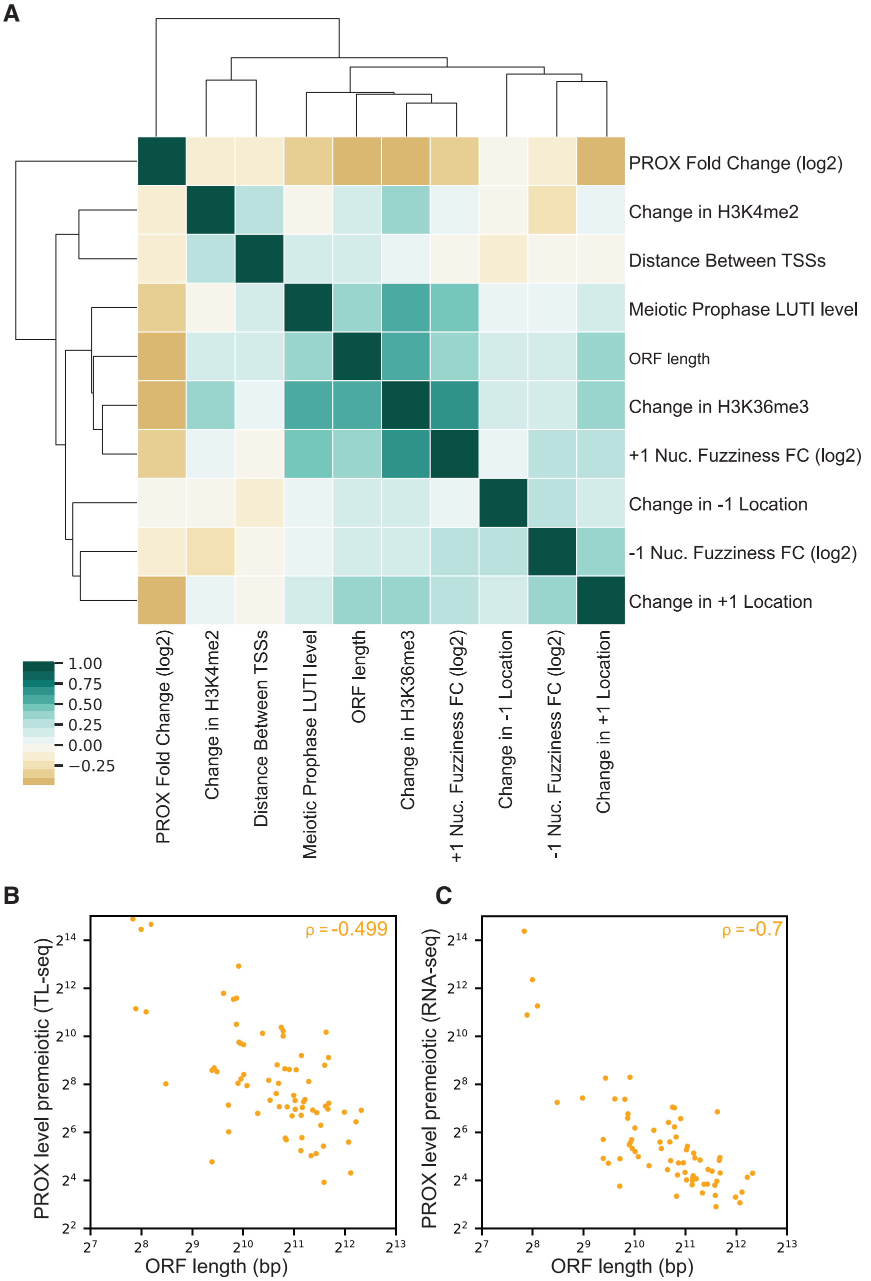

Figure 6. The key features that predict LUTI-based transcriptional repression

(A) Cluster map of possible features associated with LUTI-based repression. All of the changes to levels of chromatin modifications or features associated with nucleosomes were assessed over the PROX promoter. Features were clustered by Spearman's correlation coefficient (ρ).

(B and C) Scatterplot of the correlation between ORF (CDS) length and PROX abundance in premeiotic cells as quantified by TL-seq (B) and RNA-seq (C). The Spearman's correlation coefficient (ρ) is shown.

LUTI, an increase in H3K36me3, greater +1 nucleosome fuzziness, and an increased likelihood of being repressed by a LUTI (Figure 6A). Coincidentally, in the set of genes with meiotic LUTIs, shorter genes had higher PROX transcript abundances than did longer genes (Figures 6B and 6C). Could it be that the promoters of strongly expressed genes are better able to continue transcribing their gene products even in the presence of LUTI mRNAs? We decided to investigate this possibility further.

If a more robustly transcribed PROX isoform is resistant to repression by LUTIs, then it follows that increasing LUTI expression or decreasing PROX expression could shift the balance in favor of LUTI-based repression. To test this hypothesis, we focused on *HSP60*, a gene whose PROX isoform is highly expressed both in the premeiotic stage and in meiotic prophase. We engineered a reporter construct in which the *HSP60* regulatory region was upstream of a short-lived green fluorescent protein (ubiGFP) (Houser et al., 2012). To enable titratable expression of LUTI, a β-estradiol-inducible LexA/LexO system was used (Ottoz et al., 2014; Figure 7A). As the dose of β-estradiol increased, so did LUTI abundance and H3K36me3 over the *HSP60*^{PROX} promoter (Figures 7B and 7C). Importantly, as the expression of LUTI increased, the abundance of ubiGFP simultaneously decreased (Figures 7D and 7E). The dose-dependent decrease in ubiGFP levels was also confirmed during mitotic growth in rich media (Figures 7H and 7I). We conclude that changes to the strength of LUTI expression can lead to a decrease in protein abundance even at a strong PROX promoter.

Using mitotic cells, we further asked what would happen if both the LUTI and the PROX levels were altered. Using the same system as above, we generated a construct with a mutated Hsf1 binding site in the *HSP60*^{PROX} promoter (Figure 7F). Hsf1 is a TF that controls the expression of genes involved in heat shock response, including *HSP60* (Sakurai and Ota, 2011). Mutating the Hsf1-binding site reduced the basal expression of ubiGFP, but did not change LUTI transcript levels (Figures 7G–7I). Upon treatment with β-estradiol, cells carrying the *hsf1* mutant were highly susceptible to LUTI-based repression, decreasing to ~25% of its original level after 3 h of LUTI induction (Figures 7H and 7I). In contrast, the ubiGFP abundance reduced to only ~60% of its original level in cells carrying the wild-type *HSP60*^{PROX} promoter (Figures 7H and 7I). Thus, changes in the strength of the PROX promoter also affect LUTI-based repression.

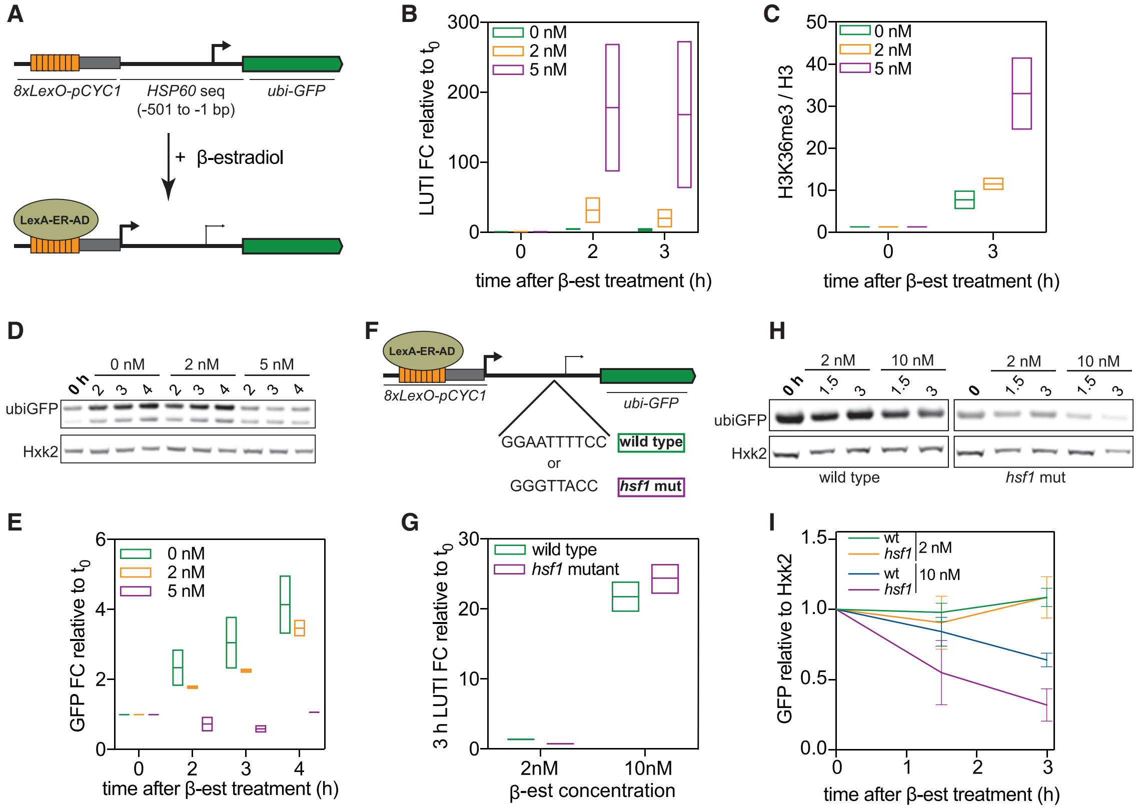

Figure 7. Changes in LUTI or PROX expression both affect LUTI-based repression of a reporter gene

(A) Illustration of a reporter construct in which the *HSP60*^{LUTI} promoter was replaced with *8xLexO* and a minimal *CYC1* promoter that can be induced upon addition of β-estradiol in cells harboring a LexA-ER-AD fusion protein. A *ubiGFP* reporter was engineered into the construct in place of the *HSP60* CDS.

(B–E) Cells with the reporter construct (*UB19257*) were induced to undergo meiosis. *HSP60-ubiGFP*^{LUTI} was induced with β-estradiol after 2 h in SPO (time 0).

(B) qRT-PCR of *HSP60-ubiGFP*^{LUTI} quantified using a primer set that spans from the region immediately upstream of the *HSP60*^{PROX} TSS until the beginning of ubiquitin. The range is displayed (n = 2).

(C) H3K36me3 ChIP was performed before induction and 3 h after induction with β-estradiol, corresponding to 5 h in SPO. Enrichment was quantified over H3 abundance using the same primer set as in (B). The range is displayed (n = 2).

(D) Immunoblot using an α-GFP antibody. Hxk2 was used as a loading control. One of 2 replicates.

(E) Immunoblot in (D) was quantified.

(F) A version of the construct in (A) was generated with a mutated Hsf1 binding site.

(G–I) Cells from the W303 background harboring the *8xLexO-HSP60-ubiGFP* construct with either a wild-type (*UB18838*) or *hsf1* (*UB20485*) mutant PROX promoter were collected during exponential mitotic growth before and after induction of *HSP60-ubiGFP*^{LUTI}.

(G) RT-qPCR of *HSP60-ubiGFP*^{LUTI}. Transcript abundance was quantified using the primer set from (B). The range is displayed (n = 2).

(H) Immunoblot using an α-GFP antibody. Hxk2 was used as a loading control. One of 2 replicates.

(I) Immunoblot was quantified relative to Hxk2 and then relative to the time of β-estradiol addition. Error bars display range (n = 2).

DISCUSSION

Developmental regulation of LUTIs

We identified 72 LUTIs in meiotic prophase. Ume6 TF was enriched in the promoter of 61 of them, with its binding motif conserved in 33 LUTIs across the *sensu stricto* genus (Figures 1D–1H). We posit that the conservation of the regulatory binding site in such a large subset of LUTI promoters indicates that they may alter gene expression in a manner that is functionally important in meiosis. By using LUTIs, a single TF can both turn on and turn off gene expression in a coordinated and timely manner to facilitate the necessary shift in proteome synthesis. We predict that LUTIs will play important roles during other developmental processes.

uORF-mediated translational inhibition of LUTIs

uORFs are ubiquitously found in walks of life from yeast to humans. Features including initiation sequence context, distance between a uORF and CDS, and uORF number can affect the degree of translational repression (Calvo et al., 2009; Chew et al., 2016; Johnstone et al., 2016). What is clear is that not all uORFs are created equal. We confirm that a single uORF can lead to repression at the *NDC80* locus, but that it depends on which uORF. The uORF closest to the LUTI TSS (uORF1) does not repress translation of the *NDC80* CDS, despite evidence from ribosome profiling that uORF1 is well translated. uORFs 5 and 9 both lead to robust repression, even though no translation of uORF 9 is observed in meiotic cells by ribosome profiling (Figure 3C; Brar et al., 2012). This

is consistent with the observation that greater distances between a uORF and CDS correlate with reduced translational repression (Chew et al., 2016; Johnstone et al., 2016). In the context of the most well-characterized case of uORF-mediated repression, *GCN4*, the distances between the 4 uORFs and the CDS start site matter greatly. That is because, upon amino acid starvation, the concentration of the ternary complex, a factor required for ribosome re-initiation, is decreased. Ultimately, the repressive uORF4 is skipped, due to extended ribosome scanning, and *GCN4* is translated (Hinnebusch, 1997). It could be worthwhile to further study the role of the ternary complex or other factors in ensuring which uORFs are integral to the translational repression of LUTIs.

The translation of uORFs and a corresponding decrease in TE is a good indication that uORFs are playing a functional role, but it does not indicate the extent of the translational regulation. TE measurements at loci with LUTIs are complicated by the presence of PROX transcripts. As an alternative to measuring TE, we measured the frequency of uORF translation. When $\geq$4 uORFs are present in a LUTI, translation occurs only 9.4% and 20.3% of the time for the penultimate and the last uORF, respectively (Figure 3D). The high correlation between PROX transcript abundance and footprints over the ORF (Figure 2E) is also a strong indication that LUTIs provide minor, if any, contribution to CDS translation. Future use of TL-seq and ribosome profiling will allow for the identification of other instances in which translational regulation is due to transcript isoform toggling rather than genuine translational regulation of a single mRNA isoform. Combined with a lack of uORF selection in the 5′ extensions of LUTIs and the increase in protein abundance upon deletion of uORFs, our study has provided conclusive evidence that uORFs in LUTIs do not just dampen CDS translation, they almost entirely repress it.

A path toward predicting LUTI-based transcriptional repression

Previous work demonstrated that only ~50% of the meiotically expressed 5′-extended transcript isoforms have poorly translated CDSs (Brar et al., 2012). If almost all 5′-extended transcripts do not translate their CDSs, then why does LUTI expression not lead to translational repression more frequently? As it turns out, robust transcriptional repression of the canonical PROX transcript by its LUTI occurs far less frequently than does translational repression of the LUTI itself. In fact, only 21 of the 72 LUTIs identified here have a corresponding decrease in the canonical PROX transcript to $\leq$25% of the starting abundance. At sites where the canonical transcript appears unchanged following LUTI expression, LUTIs may still serve a purpose. For instance, LUTI expression could prevent an upsurge in canonical mRNA levels that would otherwise occur upon meiotic entry (Figures 4I and S5A). Such a dampening may counteract unfavorable increases in protein expression.

Upon further investigation, we determined that high LUTI expression, increased H3K36me3 and nucleosome repositioning, and longer ORFs are significantly associated with LUTI-based transcriptional repression. Supporting our findings, both high upstream alternative transcription and Set2, the H3K36me3 methyltransferase, were also recently found to be associated with transcriptional repression from canonical TSSs (Chia et al., 2021). Unexpectedly, increased H3K4me2 was not associated with LUTI-based transcriptional repression, even though it is necessary for repression by $NDC80^{LUTI}$ (Chia et al., 2017). It is possible that only a subset of loci is dependent on H3K4me2 for repression, or H3K4me2 may help to delay the kinetics of PROX re-expression later in meiosis, as it does at sites of overlapping transcription (Kim et al., 2012).

Even with features correlating significantly with repression, there were still instances in which high LUTI abundance, enrichment of H3K36me3, and changes to the +1 nucleosome were observed with no apparent repression of the PROX transcript. We hypothesized that strongly expressed PROX isoforms may be more resistant to LUTIs. An in-depth analysis using a reporter construct with the *HSP60* leader demonstrated that robustly increasing LUTI levels or decreasing PROX expression can alter the sensitivity of a gene to LUTI-based repression. This supports the incorporation of PROX expression level into future analyses of LUTI-based repression.

Conclusions

In summary, we identified a group of LUTIs expressed in a coordinated manner during meiotic prophase in budding yeast. Almost all are severely translationally inhibited due to uORFs in their 5′ leaders. However, they do not ubiquitously lead to the repression of PROX promoters. Rather, an interplay between the strength of the promoters, the chromatin landscape, and possibly other yet to be discovered features interact to decide the transcriptional output. Future studies that integrate multiple datasets will help identify additional LUTIs and build predictive models to determine those that cause gene repression. In addition, it remains to be uncovered, in many cases, what the biological role of a given LUTI is and how its disruption affects cellular function. Lastly, in human cells, the presence of a LUTI at the *MDM2* locus (Hollerer et al., 2019) opens the door for more studies into how significant LUTIs are in human development and disease.

Limitations of study

Here, we uncover examples whereby the upstream transcription of a LUTI, unexpectedly, does not alter the gene expression of a downstream promoter. Our analyses identified correlative relationships between PROX repression, LUTI expression, and changes to the chromatin landscape over the PROX promoter. A firm understanding of the causative interactions between such features is not addressed and is a key limitation. In addition, while we investigate the relationship between the LUTI and PROX promoters at the *HSP60* locus in some depth, a more detailed exploration of LUTI and PROX promoter strength modulation under additional contexts has the potential to improve our understanding of the complex interplay that occurs when a single gene is controlled by the expression of transcripts from two distinct promoters. Lastly, we demonstrate that during early meiosis, LUTI expression occurs in a developmentally regulated fashion, but we have not yet established the biological need for such timely LUTI expression.

CellPress
OPEN ACCESS

STAR★METHODS

Detailed methods are provided in the online version of this paper and include the following:

- KEY RESOURCES TABLE
- RESOURCE AVAILABILITY
  - Lead contact
  - Materials availability
  - Data and code availability
- EXPERIMENTAL MODEL AND SUBJECT DETAILS
  - Strain construction and cloning
  - *pCUP1-IME1/pCUP1-IME4* sporulation
  - Mitotic cell collection
- METHOD DETAILS
  - RNA extraction for TL-seq, nanopore sequencing and RNA-seq
  - Transcript leader sequencing (TL-seq)
  - polyA selection for Nanopore sequencing and RNA-seq
  - Nanopore sequencing
  - Chromatin immunoprecipitation (ChIP)
  - RNA-seq
  - Immunoblotting
  - RNA extraction for blotting and RT-qPCR
  - RNA blotting
  - Micrococcal nuclease sequencing (MNase-seq)
  - Reverse transcription-quantitative polymerase chain reaction (RT-qPCR)
- QUANTIFICATION AND STATISTICAL ANALYSIS
  - TL-seq analysis
  - Pipeline for 5′-extended transcript discovery
  - Motif discovery
  - Ume6 ChIP-seq analysis
  - Conservation of URS1 binding sites
  - Gene set enrichment analysis (GSEA)
  - uORF analysis
  - Chromatin modification analysis
  - RNA-seq analysis
  - MNase-seq analysis
  - Clustered heatmap

SUPPLEMENTAL INFORMATION

Supplemental information can be found online at https://doi.org/10.1016/j.molcel.2021.03.013.

ACKNOWLEDGMENTS

We thank Nicholas Ingolia, Gloria Brar, and Cai Li for their help with the data analysis; Michael Eisen, Nicholas Ingolia, Gloria Brar, Jasper Rine, Jingxun Chen, Tina Sing, Amanda Su, Eric Sawyer, Grant King, and Andrea Higdon for suggestions and comments on this manuscript; and Tina Sing and Anthony Harris for technical support. This work is supported by funds from the Curci Foundation, the Pew Charitable Trusts (00027344), and the National Institutes of Health (NIH, DP2 AG055946-01 and R01 GM140005) to E.Ü. F.J.v.W. and M.C. are supported by the Francis Crick Institute, which receives its core funding from Cancer Research UK (FC001203), the UK Medical Research Council (FC001203), and the Wellcome Trust (FC001203). M.C. is supported by an A*STAR scholarship. K.M. is supported by an NIH F31HD103399 fellowship.

AUTHOR CONTRIBUTIONS

A.T., conceptualization, data curation, formal analysis, investigation, methodology, validation, visualization, and drafting of the manuscript. K.M., data curation, formal analysis, investigation, validation, manuscript editing, and visualization. V.J., data curation, formal analysis, investigation, methodology, validation, and visualization. M.C., TL-seq library preparations and manuscript editing. H.L., investigation of the *NDC80* uORF analysis. F.J.v.W., supervision, project administration, and manuscript editing. E.Ü., conceptualization, formal analysis, methodology, visualization, supervision, project administration, funding acquisition, and drafting of the manuscript.

DECLARATION OF INTERESTS

The authors declare no competing interests.

Received: November 8, 2019
Revised: February 5, 2021
Accepted: March 9, 2021
Published: April 6, 2021

REFERENCES

Arribere, J.A., and Gilbert, W.V. (2013). Roles for transcript leaders in translation and mRNA decay revealed by transcript leader sequencing. Genome Res. *23*, 977–987.

Asakawa, H., Hayashi, A., Haraguchi, T., and Hiraoka, Y. (2005). Dissociation of the Nuf2-Ndc80 complex releases centromeres from the spindle-pole body during meiotic prophase in fission yeast. Mol. Biol. Cell *16*, 2325–2338.

Ashburner, M., Ball, C.A., Blake, J.A., Botstein, D., Butler, H., Cherry, J.M., Davis, A.P., Dolinski, K., Dwight, S.S., Eppig, J.T., et al.; The Gene Ontology Consortium (2000). Gene ontology: tool for the unification of biology. Nat. Genet. *25*, 25–29.

Bailey, T.L., and Elkan, C. (1994). Fitting a mixture model by expectation maximization to discover motifs in biopolymers. Proc. Int. Conf. Intell. Syst. Mol. Biol. *2*, 28–36.

Batut, P., Dobin, A., Plessy, C., Carninci, P., and Gingeras, T.R. (2013). High-fidelity promoter profiling reveals widespread alternative promoter usage and transposon-driven developmental gene expression. Genome Res. *23*, 169–180.

Berchowitz, L.E., Gajadhar, A.S., van Werven, F.J., De Rosa, A.A., Samoylova, M.L., Brar, G.A., Xu, Y., Xiao, C., Futcher, B., Weissman, J.S., et al. (2013). A developmentally regulated translational control pathway establishes the meiotic chromosome segregation pattern. Genes Dev. *27*, 2147–2163.

Bowdish, K.S., Yuan, H.E., and Mitchell, A.P. (1995). Positive control of yeast meiotic genes by the negative regulator UME6. Mol. Cell. Biol. *15*, 2955–2961.

Brar, G.A., Yassour, M., Friedman, N., Regev, A., Ingolia, N.T., and Weissman, J.S. (2012). High-resolution view of the yeast meiotic program revealed by ribosome profiling. Science *335*, 552–557.

Calvo, S.E., Pagliarini, D.J., and Mootha, V.K. (2009). Upstream open reading frames cause widespread reduction of protein expression and are polymorphic among humans. Proc. Natl. Acad. Sci. USA *106*, 7507–7512.

Carbon, S., Douglass, E., Good, B.M., Unni, D.R., Harris, N.L., Mungall, C.J., Basu, S., Chisholm, R.L., Dodson, R.J., Hartline, E., et al.; Gene Ontology Consortium (2021). The Gene Ontology resource: enriching a GOld mine. Nucleic Acids Res. *49* (D1), D325–D334.

Carlile, T.M., and Amon, A. (2008). Meiosis I is established through division-specific translational control of a cyclin. Cell *133*, 280–291.

Chen, K., Xi, Y., Pan, X., Li, Z., Kaestner, K., Tyler, J., Dent, S., He, X., and Li, W. (2013). DANPOS: dynamic analysis of nucleosome position and occupancy by sequencing. Genome Res. *23*, 341–351.

Chen, J., Tresenrider, A., Chia, M., McSwiggen, D.T., Spedale, G., Jorgensen, V., Liao, H., van Werven, F.J., and Ünal, E. (2017). Kinetochore inactivation by expression of a repressive mRNA. eLife *6*, e27417.

Cheng, Z., Otto, G.M., Powers, E.N., Keskin, A., Mertins, P., Carr, S.A., Jovanovic, M., and Brar, G.A. (2018). Pervasive, Coordinated Protein-Level Changes Driven by Transcript Isoform Switching during Meiosis. Cell *172*, 910–923.e16.

Chew, G.L., Pauli, A., and Schier, A.F. (2016). Conservation of uORF repressiveness and sequence features in mouse, human and zebrafish. Nat. Commun. *7*, 11663.

Chia, M., and van Werven, F.J. (2016). Temporal Expression of a Master Regulator Drives Synchronous Sporulation in Budding Yeast. G3 (Bethesda) *6*, 3553–3560.

Chia, M., Tresenrider, A., Chen, J., Spedale, G., Jorgensen, V., Ünal, E., and van Werven, F.J. (2017). Transcription of a 5′ extended mRNA isoform directs dynamic chromatin changes and interference of a downstream promoter. eLife *6*, e27420.

Chia, M., Li, C., Marques, S., Pelechano, V., Luscombe, N.M., and van Werven, F.J. (2021). High-resolution analysis of cell-state transitions in yeast suggests widespread transcriptional tuning by alternative starts. Genome Biol. *22*, e34.

Dobin, A., Davis, C.A., Schlesinger, F., Drenkow, J., Zaleski, C., Jha, S., Batut, P., Chaisson, M., and Gingeras, T.R. (2013). STAR: ultrafast universal RNA-seq aligner. Bioinformatics *29*, 15–21.

Garalde, D.R., Snell, E.A., Jachimowicz, D., Sipos, B., Lloyd, J.H., Bruce, M., Pantic, N., Admassu, T., James, P., Warland, A., et al. (2018). Highly parallel direct RNA sequencing on an array of nanopores. Nat. Methods *15*, 201–206.

Gibson, D.G., Young, L., Chuang, R.-Y., Venter, J.C., Hutchison, C.A., 3rd, and Smith, H.O. (2009). Enzymatic assembly of DNA molecules up to several hundred kilobases. Nat. Methods *6*, 343–345.

Haberle, V., Forrest, A.R.R., Hayashizaki, Y., Carninci, P., and Lenhard, B. (2015). CAGEr: precise TSS data retrieval and high-resolution promoterome mining for integrative analyses. Nucleic Acids Res. *43*, e51.

Hinnebusch, A.G. (1997). Translational regulation of yeast GCN4. A window on factors that control initiator-trna binding to the ribosome. J. Biol. Chem. *272*, 21661–21664.

Hinnebusch, A.G., Ivanov, I.P., and Sonenberg, N. (2016). Translational control by 5′-untranslated regions of eukaryotic mRNAs. Science *352*, 1413–1416.

Hollerer, I., Barker, J.C., Jorgensen, V., Tresenrider, A., Dugast-Darzacq, C., Chan, L.Y., Darzacq, X., Tjian, R., Ünal, E., and Brar, G.A. (2019). Evidence for an Integrated Gene Repression Mechanism Based on mRNA Isoform Toggling in Human Cells. G3 (Bethesda) *9*, 1045–1053.

Houser, J.R., Ford, E., Chatterjea, S.M., Maleri, S., Elston, T.C., and Errede, B. (2012). An improved short-lived fluorescent protein transcriptional reporter for Saccharomyces cerevisiae. Yeast *29*, 519–530.

Hunter, J.D. (2007). Matplotlib: A 2D graphics environment. Comput. Sci. Eng. *9*, 99–104.

Ingolia, N.T., Ghaemmaghami, S., Newman, J.R.S., and Weissman, J.S. (2009). Genome-wide analysis in vivo of translation with nucleotide resolution using ribosome profiling. Science *324*, 218–223.

Johnstone, T.G., Bazzini, A.A., and Giraldez, A.J. (2016). Upstream ORFs are prevalent translational repressors in vertebrates. EMBO J. *35*, 706–723.

Kent, W.J., Zweig, A.S., Barber, G., Hinrichs, A.S., and Karolchik, D. (2010). BigWig and BigBed: enabling browsing of large distributed datasets. Bioinformatics *26*, 2204–2207.

Kim, J.H., Lee, B.B., Oh, Y.M., Zhu, C., Steinmetz, L.M., Lee, Y., Kim, W.K., Lee, S.B., Buratowski, S., and Kim, T. (2016). Modulation of mRNA and lncRNA expression dynamics by the Set2-Rpd3S pathway. Nat. Commun. *7*, 13534.

Kim, T., Xu, Z., Clauder-Munster, S., Steinmetz, L.M., and Buratowski, S. (2012). Set3 HDAC mediates effects of overlapping noncoding transcription on gene induction kinetics. Cell *150*, 1158–1169.

Kimura, K., Wakamatsu, A., Suzuki, Y., Ota, T., Nishikawa, T., Yamashita, R., Yamamoto, J., Sekine, M., Tsuritani, K., Wakaguri, H., et al. (2006). Diversification of transcriptional modulation: large-scale identification and characterization of putative alternative promoters of human genes. Genome Res. *16*, 55–65.

Klemm, S.L., Shipony, Z., and Greenleaf, W.J. (2019). Chromatin accessibility and the regulatory epigenome. Nat. Rev. Genet. *20*, 207–220.

Koster, M.J.E., Yildirim, A.D., Weil, P.A., Holstege, F.C.P., and Timmers, H.T.M. (2014). Suppression of intragenic transcription requires the MOT1 and NC2 regulators of TATA-binding protein. Nucleic Acids Res. *42*, 4220–4229.

Langmead, B., and Salzberg, S.L. (2012). Fast gapped-read alignment with Bowtie 2. Nat. Methods *9*, 357–359.

Langmead, B., Wilks, C., Antonescu, V., and Charles, R. (2019). Scaling read aligners to hundreds of threads on general-purpose processors. Bioinformatics *35*, 421–432.

Li, H. (2018). Minimap2: pairwise alignment for nucleotide sequences. Bioinformatics *34*, 3094–3100.

Love, M.I., Huber, W., and Anders, S. (2014). Moderated estimation of fold change and dispersion for RNA-seq data with DESeq2. Genome Biol. *15*, 550.

Malabat, C., Feuerbach, F., Ma, L., Saveanu, C., and Jacquier, A. (2015). Quality control of transcription start site selection by nonsense-mediated-mRNA decay. eLife *4*, e06722.

Martin, M. (2011). Cutadapt removes adapter sequences from high-throughput sequencing reads. EMBnet. J. *17*, 10–12.

Miller, M.P., Unal, E., Brar, G.A., and Amon, A. (2012). Meiosis I chromosome segregation is established through regulation of microtubule-kinetochore interactions. eLife *1*, e00117.

Mootha, V.K., Lindgren, C.M., Eriksson, K.-F., Subramanian, A., Sihag, S., Lehar, J., Puigserver, P., Carlsson, E., Ridderstråle, M., Laurila, E., et al. (2003). PGC-1α-responsive genes involved in oxidative phosphorylation are coordinately downregulated in human diabetes. Nat. Genet. *34*, 267–273.

Ottoz, D.S.M., Rudolf, F., and Stelling, J. (2014). Inducible, tightly regulated and growth condition-independent transcription factor in Saccharomyces cerevisiae. Nucleic Acids Res. *42*, e130.

Pagè, H. (2018). BSgenome: Software infrastructure for efficient representation of full genomes and their SNPs. https://rdrr.io/bioc/BSgenome/.

Park, H.D., Luche, R.M., and Cooper, T.G. (1992). The yeast UME6 gene product is required for transcriptional repression mediated by the CAR1 URS1 repressor binding site. Nucleic Acids Res. *20*, 1909–1915.

Patro, R., Duggal, G., Love, M.I., Irizarry, R.A., and Kingsford, C. (2017). Salmon provides fast and bias-aware quantification of transcript expression. Nat. Methods *14*, 417–419.

Quinlan, A.R., and Hall, I.M. (2010). BEDTools: a flexible suite of utilities for comparing genomic features. Bioinformatics *26*, 841–842.

Ramírez, F., Ryan, D.P., Grüning, B., Bhardwaj, V., Kilpert, F., Richter, A.S., Heyne, S., Dündar, F., and Manke, T. (2016). deepTools2: a next generation web server for deep-sequencing data analysis. Nucleic Acids Res. *44* (W1), W160–W165.

Rodriguez, J., McKnight, J.N., and Tsukiyama, T. (2014). Genome-Wide Analysis of Nucleosome Positions, Occupancy, and Accessibility in Yeast: Nucleosome Mapping, High-Resolution Histone ChIP, and NCAM. Curr. Protoc. Mol. Biol. *2014*, 21.28.1–21.28.16.

Sakurai, H., and Ota, A. (2011). Regulation of chaperone gene expression by heat shock transcription factor in Saccharomyces cerevisiae: importance in normal cell growth, stress resistance, and longevity. FEBS Lett. *585*, 2744–2748.

Siepel, A., Bejerano, G., Pedersen, J.S., Hinrichs, A.S., Hou, M., Rosenbloom, K., Clawson, H., Spieth, J., Hillier, L.W., Richards, S., et al. (2005). Evolutionarily conserved elements in vertebrate, insect, worm, and yeast genomes. Genome Res. *15*, 1034–1050.

Subramanian, A., Tamayo, P., Mootha, V.K., Mukherjee, S., Ebert, B.L., Gillette, M.A., Paulovich, A., Pomeroy, S.L., Golub, T.R., Lander, E.S., and Mesirov, J.P. (2005). Gene set enrichment analysis: a knowledge-based approach for interpreting genome-wide expression profiles. Proc. Natl. Acad. Sci. USA *102*, 15545–15550.

CellPress
OPEN ACCESS

Sumrada, R.A., and Cooper, T.G. (1987). Ubiquitous upstream repression sequences control activation of the inducible arginase gene in yeast. Proc. Natl. Acad. Sci. USA *84*, 3997–4001.

Sun, S.-C., Zhang, D.-X., Lee, S.-E., Xu, Y.-N., and Kim, N.-H. (2011). Ndc80 regulates meiotic spindle organization, chromosome alignment, and cell cycle progression in mouse oocytes. Microsc. Microanal. *17*, 431–439.

Van Dalfsen, K.M., Hodapp, S., Keskin, A., Otto, G.M., Berdan, C.A., Higdon, A., Cheunkarndee, T., Nomura, D.K., Jovanovic, M., and Brar, G.A. (2018). Global Proteome Remodeling during ER Stress Involves Hac1-Driven Expression of Long Undecoded Transcript Isoforms. Dev. Cell *46*, 219–235.e8.

Venkatesh, S., and Workman, J.L. (2015). Histone exchange, chromatin structure and the regulation of transcription. Nat. Rev. Mol. Cell Biol. *16*, 178–189.

Williams, R.M., Primig, M., Washburn, B.K., Winzeler, E.A., Bellis, M., Sarrauste de Menthiere, C., Davis, R.W., and Esposito, R.E. (2002). The Ume6 regulon coordinates metabolic and meiotic gene expression in yeast. Proc. Natl. Acad. Sci. USA *99*, 13431–13436.

Wu, A.C.K., Patel, H., Chia, M., Moretto, F., Frith, D., Snijders, A.P., and van Werven, F.J. (2018). Repression of divergent noncoding transcription by a sequence-specific transcription factor. Mol. Cell *72*, 942–54.e947.

Yue, J.X., Li, J., Aigrain, L., Hallin, J., Persson, K., Oliver, K., Bergström, A., Coupland, P., Warringer, J., Lagomarsino, M.C., et al. (2017). Contrasting evolutionary genome dynamics between domesticated and wild yeasts. Nat. Genet. *49*, 913–924.

Zhang, Y., Liu, T., Meyer, C.A., Eeckhoute, J., Johnson, D.S., Bernstein, B.E., Nusbaum, C., Myers, R.M., Brown, M., Li, W., and Liu, X.S. (2008). Model-based analysis of ChIP-Seq (MACS). Genome Biol. *9*, R137.

STAR★METHODS

KEY RESOURCES TABLE

REAGENT or RESOURCE	SOURCE	IDENTIFIER
**Antibodies**		
H3K36me3	abcam	ab9050, RRID:AB_306966
H3K4me2	abcam	ab32356, RRID:AB_7329224
mouse α-V5	Thermo Fisher	R960-25, RRID:AB_2556564
α-GFP	Takara	632381, RRID:AB_2313808
rabbit α-hexokinase (α-Hxk2)	US Biological	H2035-01, RRID:AB_2629457
α-mouse antibody conjugated to IRDye 800CW	LI-COR Biosciences	926-32212, RRID:AB_621847
α-rabbit antibody conjugated to IRDye 680RD	LI-COR Biosciences	926-68071, RRID:AB_10956166
**Chemicals, peptides, and recombinant proteins**		
Zinc-based alkaline fragmentation reagent	Ambion	AM8740
recombinant shrimp alkaline phosphatase	NEW ENGLAND BIOLABS	M0371
RNasin Plus	Promega	N2611
Cap-Clip acid pyrophosphatase	Tebu-Bio	C-CC15011H
T4 RNA ligase 1	NEW ENGLAND BIOLABS	M0437M
SuperScript IV reverse transcriptase	Invitrogen	18090010
RNase H	NEW ENGLAND BIOLABS	M0297
RNase cocktail enzyme mix	Ambion	AM2286
HighPrep PCR beads	MagBio	AC-60050
KAPA Hi-Fi hot start ready mix	Roche	KK2601
KAPA single indexed adapters Set B	Roche	KK8702
MyOne Streptavidin C1 Dynabeads	ThermoFisher Scientific	65001
oligo-dT DynaBeads	ThermoFisher Scientific	61002
R9.4.1 flow cell	Oxford Nanopore Technologies	FLO-MIN106
Protein A Dynabeads	Invitrogen	10001D
RNase A	Invitrogen	12091021
Proteinase K	Roche	3115879001
AMPure XP beads	Beckman Coulter	A63881
ULTRAhyb Ultrasensitive Hybridization Buffer	ThermoFisher Scientific	AM8669
Exonuclease III	New England Biolabs	M0206S
Mnase	Worthington	LS004797
Superscript III kit	ThermoFisher Scientific	18080044
Absolute Blue qPCR Mix	ThermoFisher Scientific	AB4162B
**Critical commercial assays**		
Poly(A)Purist MAG kit	Ambion	AM1922
KAPA Hyper Prep Kit	Roche	KK8504
Direct RNA Sequencing Kit	Oxford Nanopore Technologies	SQK-RNA001
ThruPLEX DNA-seq Kit	Takara	R400427
NEXTflex^{TM} Rapid Directional mRNA-Seq Kit	Bioo Scientific	NOVA-5138
Prime-It II Random Primer Labeling Kit	Agilent Technologies, Inc	300385

(*Continued on next page*)

CellPress
OPEN ACCESS

Continued

REAGENT or RESOURCE	SOURCE	IDENTIFIER
Deposited data		
TL-seq, RNA-seq, Direct RNA Nanopore Sequencing, ChIP-seq, MNase-seq	This study	GEO: GSE140177
RNA-seq, Ribosome Profiling	Cheng et al. (2018)	GEO: GSE108778
Experimental models: organisms/strains		
S. cerevisiae: UB3301: MATa /MATalpha UME6-3V5::His3MX/UME6-3V5::His3MX irt1:cup1::Hphmx/irt1:cup1::Hphmx ime4::cup1::NAT/ime4::cup1::NAT	Brar-Ünal lab	N/A
S. cerevisiae: UB6183: MATa /MATalpha irt1:cup1::Hphmx/irt1:cup1::Hphmx ime4::cup1::NAT/ime4::cup1::NAT leu2::Δ9AUG-NDC80-3V5:LEU2/ leu2::Δ9AUG-NDC80-3V5:LEU2 ndc80Δ(−1000 and ORF):KanMX4 /ndc80Δ(−1000 and ORF):KanMX4	Brar-Ünal lab	N/A
S. cerevisiae: UB6190: MATa /MATalpha irt1:cup1::Hphmx/irt1:cup1::Hphmx ime4::cup1::NAT/ime4::cup1::NAT ndc80Δ(−1000 and ORF):KanMX4 /ndc80Δ(−1000 and ORF):KanMX4 leu2::NDC80-3V5:LEU2/leu2::NDC80-3V5:LEU2	Brar-Ünal lab	N/A
S. cerevisiae: UB10579: MATa/MATalpha irt1:cup1::Hphmx/irt1:cup1::Hphmx ime4::cup1::NAT/ime4::cup1::NAT leu2::pUB88-Δ8AUG-uORF1-revert-5′UTR-NDC80-3V5:LEU2/leu2::pUB88-Δ8AUG-uORF1-revert-5′UTR-NDC80-3V5:LEU2 ndc80Δ(−1000 and ORF):KanMX4/ndc80Δ(−1000 and ORF):KanMX4	This study	N/A
S. cerevisiae: UB10581: MATa/MATalpha irt1:cup1::Hphmx/irt1:cup1::Hphmx ime4::cup1::NAT/ime4::cup1::NAT leu2::pUB88-Δ8AUG-uORF5-revert-5′UTR-NDC80-3V5:LEU2/leu2::pUB88-Δ8AUG-uORF5-revert-5′UTR-NDC80-3V5:LEU2 ndc80Δ(−1000 and ORF):KanMX4/ndc80Δ(−1000 and ORF):KanMX4	This study	N/A
S. cerevisiae: UB10583: MATa/MATalpha irt1:cup1::Hphmx/irt1:cup1::Hphmx ime4::cup1::NAT/ime4::cup1::NAT leu2::pUB88-Δ8AUG-uORF9-revert-5′UTR-NDC80-3V5:LEU2/leu2::pUB88-Δ8AUG-uORF9-revert-5′UTR-NDC80-3V5:LEU2 ndc80Δ(−1000 and ORF):KanMX4/ndc80Δ(−1000 and ORF):KanMX4	This study	N/A
S. cerevisiae: UB14584: MATa/MATalpha pCUP-IME1::NAT/pCUP-IME1::NAT pCUP-IME4::NAT/pCUP-IME4::NAT amn1(BY4741 allele)unmarked/ amn1(BY4741 allele)unmarked	This study	N/A

(*Continued on next page*)

Continued

REAGENT or RESOURCE	SOURCE	IDENTIFIER
S. cerevisiae: UB18175: MATa/MATalpha irt1:cup1::Hphmx/irt1:cup1::Hphmx ime4::cup1::NAT/ime4::cup1::NAT leu2::SWI4-3V5-NDC80term::LEU2/ leu2::SWI4-3V5-NDC80term::LEU2	This study	N/A
S. cerevisiae: UB18176: MATa/MATalpha irt1:cup1::Hphmx/irt1:cup1::Hphmx ime4::cup1::NAT/ime4::cup1::NAT leu2::pSWI4LUTI(−1200 to −934)Δ-SWI4-3V5-NDC80term::LEU2/ leu2::pSWI4LUTI(−1200 to −934)Δ-SWI4-3V5-NDC80term::LEU2	This study	N/A
S. cerevisiae: UB18181: MATa/MATalpha irt1:cup1::Hphmx/irt1:cup1::Hphmx ime4::cup1::NAT/ime4::cup1::NAT leu2::pAPL4LUTIΔ(−415 to −800)-APL4-3V5-NDC80term::LEU2/ leu2::pAPL4Δ(−415 to −800)-APL4-3V5-NDC80term::LEU2	This study	N/A
S. cerevisiae: UB18185: MATa/MATalpha irt1:cup1::Hphmx/irt1:cup1::Hphmx ime4::cup1::NAT/ime4::cup1::NAT leu2::CDC60-3V5-NDC80term::LEU2/ leu2::CDC60-3V5-NDC80term::LEU2	This study	N/A
S. cerevisiae: UB18186: MATa/MATalpha irt1:cup1::Hphmx/irt1:cup1::Hphmx ime4::cup1::NAT/ime4::cup1::NAT leu2::pCDC60LUTI(−1000 to −483)Δ-CDC60-3V5-NDC80term::LEU2/ leu2::pCDC60LUTI(−1000 to −483)Δ-CDC60-3V5-NDC80term::LEU2	This study	N/A
S. cerevisiae: UB18188: MATa/MATalpha irt1:cup1::Hphmx/irt1:cup1::Hphmx ime4::cup1::NAT/ime4::cup1::NAT leu2::pHSP60LUTIΔ(−1000 to −501)-HSP60-3V5-NDC80term::LEU2/ leu2::pHSP60Δ(−1000 to −501)-HSP60-3V5-NDC80term::LEU2	This study	N/A
S. cerevisiae: UB18190: MATa/MATalpha irt1:cup1::Hphmx/irt1:cup1::Hphmx ime4::cup1::NAT/ime4::cup1::NAT leu2::pMSC6LUTI(−800 to −335)Δ-MSC6-3V5-NDC80term::LEU2/ leu2::pMSC6LUTI(−800 to −335)Δ-MSC6-3V5-NDC80term::LEU2	This study	N/A
S. cerevisiae: UB18238: MATa/MATalpha irt1:cup1::Hphmx/irt1:cup1::Hphmx ime4::cup1::NAT/ime4::cup1::NAT leu2::MSC6-3V5-NDC80term::LEU2/ leu2::MSC6-3V5-NDC80term::LEU2	This study	N/A
S. cerevisiae: UB18336: MATa/MATalpha irt1:cup1::Hphmx/irt1:cup1::Hphmx ime4::cup1::NAT/ime4::cup1::NAT leu2::HSP60-3V5-NDC80term::LEU2/ leu2::HSP60-3V5-NDC80term::LEU2	This study	N/A

(*Continued on next page*)

CellPress
OPEN ACCESS

Continued

REAGENT or RESOURCE	SOURCE	IDENTIFIER
S. cerevisiae: UB18539: MATa/MATalpha irt1:cup1::Hphmx/irt1:cup1::Hphmx ime4::cup1::NAT/ime4::cup1::NAT leu2::APL4-3V5-NDC80term::LEU2/ leu2::APL4-3V5-NDC80term::LEU2	This study	N/A
S. cerevisiae: UB18838: MATa trp1::pGPD1-LexA-ER-HA-B112::TRP1 his3::8xLexO-HSP60leader-ubiGFP::HIS3	This study	N/A
S. cerevisiae: UB19257: MATa/MATalpha trp1::pGPD1-LexA-ER-HA-B112::TRP1/ trp1::pGPD1-LexA-ER-HA-B112::TRP1 irt1:cup1::Hphmx/irt1:cup1::Hphmx ime4::cup1::NAT/ime4::cup1::NAT his3::8xLexO-HSP60leader-ubiGFP::HIS3/ his3::8xLexO-HSP60leader-ubiGFP::HIS3	This study	N/A
S. cerevisiae: UB20485: MATa trp1::pGPD1-LexA-ER-HA-B112::TRP1 his3::8xLexO-hsf1-mut1-HSP60leader-ubiGFP::HIS3	This study	N/A
S. cerevisiae: UB20649: MATa/MATalpha irt1::pCUP1-3HA-IME1::KANMX/ irt1::pCUP1-3HA-IME1::KANMX ime4::pCUP1-3HA-IME4::KANMX/ ime4::pCUP1-3HA-IME4::KANMX Ume6-3v5::His3MX/Ume6-3v5::His3MX	This study	N/A
S. cerevisiae: UB22629: MATa/MATalpha irt1::pCUP1-3HA-IME1::KANMX/ irt1::pCUP1-3HA-IME1::KANMX pCUP-IME4::NAT/pCUP-IME4::NAT ume6::UME6(T99N)-3V5::KanMX/ ume6::UME6(T99N)-3V5::KanMX	This study	N/A
S. cerevisiae: UB26122: MATa/MATalpha irt1:cup1::Hphmx/irt1:cup1::Hphmx ime4::cup1::NAT/ime4::cup1::NAT leu2::APL4uORFdel-3V5-NDC80term::LEU2/leu2::APL4uORFdel-3V5-NDC80term::LEU2	This study	N/A
S. cerevisiae: UB26123: MATa/MATalpha irt1:cup1::Hphmx/irt1:cup1::Hphmx ime4::cup1::NAT/ime4::cup1::NAT leu2::HSP60uORFdel-3V5-NDC80term::LEU2/leu2::HSP60uORFdel-3V5-NDC80term::LEU2	This study	N/A
S. cerevisiae: UB26124: MATa/MATalpha irt1:cup1::Hphmx/irt1:cup1::Hphmx ime4::cup1::NAT/ime4::cup1::NAT leu2::MSC6uORFdel-3V5-NDC80term::LEU2/leu2::MSC6uORFdel-3V5-NDC80term::LEU2	This study	N/A
S. cerevisiae: UB26705: MATa/MATalpha irt1:cup1::Hphmx/irt1:cup1::Hphmx ime4::cup1::NAT/ime4::cup1::NAT leu2::HSP60-3V5-NDC80term::LEU2/ leu2::HSP60-3V5-NDC80term::LEU2 upf1::NAT/upf1::NAT	This study	N/A

(*Continued on next page*)

Continued

REAGENT or RESOURCE	SOURCE	IDENTIFIER
S. cerevisiae: UB26707: MATa/MATalpha irt1:cup1::Hphmx/irt1:cup1::Hphmx ime4::cup1::NAT/ime4::cup1::NAT leu2::APL4-3V5-NDC80term::LEU2/ leu2::APL4-3V5-NDC80term::LEU2 upf1::NAT/upf1::NAT	This study	N/A
S. cerevisiae: UB26708: MATa/MATalpha irt1:cup1::Hphmx/irt1:cup1::Hphmx ime4::cup1::NAT/ime4::cup1::NAT leu2::MSC6-3V5NDC80term::LEU2/ leu2::MSC6-3V5-NDC80term::LEU2 upf1::NAT/upf1::NAT	This study	N/A
Oligonucleotides		
3V5 probe primers	See Table S7	N/A
ACT1 qPCR primers	See Table S7	N/A
PFY1 qPCR primers	See Table S7	N/A
HSP60/UBI qPCR primers	See Table S7	N/A
APL4 qPCR primers	See Table S7	N/A
HSP60 qPCR primers	See Table S7	N/A
MSC6 qPCR primers	See Table S7	N/A
Software and algorithms		
cutadapt, v2.3	Martin, 2011	https://cutadapt.readthedocs.io/en/stable/
STAR, v2.5.3a	Dobin et al., 2013	https://github.com/alexdobin/STAR
BSgenome, v1.50.0	Pagè, 2018	https://bioconductor.org/packages/release/bioc/html/BSgenome.html
CAGEr, v1.24.0	Haberle et al., 2015	https://bioconductor.org/packages/release/bioc/html/CAGEr.html
DESeq2, v1.22.2	Love et al., 2014	https://bioconductor.org/packages/release/bioc/html/DESeq2.html
MinKNOW, v1.10.23	Oxford Nanopore Technologies	https://nanoporetech.com/
Albacore, v2.1.10	Oxford Nanopore Technologies	https://nanoporetech.com/
minimap2, v2.9-r720	Li, 2018	https://github.com/lh3/minimap2
Meme	Bailey and Elkan, 1994	https://meme-suite.org/tools/meme
bowtie2, v2.3.4.3	Langmead and Salzberg, 2012	http://bowtie-bio.sourceforge.net/bowtie2/index.shtml
macs2, v2.1.1.20160309	Zhang et al., 2008	https://github.com/macs3-project/MACS
bedGraphToBigWig, v4	Kent et al., 2010	https://bioconda.github.io/recipes/ucsc-bedgraphtobigwig/README.html
deeptools2, v3.0.1	Ramírez et al., 2016	https://deeptools.readthedocs.io/en/develop/
phastCons	Siepel et al., 2005	http://compgen.cshl.edu/phast/phastCons-HOWTO.html
salmon, v0.13.1	Patro et al., 2017	https://combine-lab.github.io/salmon/
matplotlib	Hunter, 2007	https://matplotlib.org/
Image Studio Lite	LI-COR	https://www.licor.com/bio/image-studio-lite/
Graphpad Prism	GraphPad Software	https://www.graphpad.com/scientific-software/prism/
bedtools	Quinlan and Hall, 2010	https://bedtools.readthedocs.io/en/latest/
GSEA, v4.1.0	Subramanian et al., 2005 and Mootha et al., 2003	https://www.gsea-msigdb.org/gsea/index.jsp
DANPOS2, v2.2.2	Chen et al., 2013	https://sites.google.com/site/danposdoc/

(*Continued on next page*)

Continued

REAGENT or RESOURCE	SOURCE	IDENTIFIER
custom code	this study	https://github.com/atresen/LUTI_key_features
Other		
minION	Oxford Nanopore Technologies	MIN-101B
Bioruptor Pico	Diagenode	B01060010

RESOURCE AVAILABILITY

Lead contact

The Lead Contact, Elçin Ünal (elcin@berkeley.edu), can provide further information and fulfill resource requests.

Materials availability

All yeast strains generated in this study are available and can be requested by contacting the Lead Contact.

Data and code availability

Data generated in this study are available at NCBI GEO under the accession ID GSE140177 (https://www.ncbi.nlm.nih.gov/geo/query/acc.cgi?acc=GSE140177). The custom code used for the analysis is available in the following code repository: https://github.com/atresen/LUTI_key_features.

EXPERIMENTAL MODEL AND SUBJECT DETAILS

Strain construction and cloning

All strains for the meiotic experiments were derived from the SK1 background and contained the previously published *pCUP1-IME1*/*pCUP1-IME4* meiotic synchronization system (Berchowitz et al., 2013). The genotypes of all the strains used in this study are listed in the Key resources table. For LUTI-regulated genes of interest (*SWI4*, *APL4*, *CDC60*, *HSP60*, and *MSC6*), the wild-type gene with between 800 and 1200 bp upstream of the CDS, depending on the length of the 5′-extension, and a C-terminal 3V5 epitope tag with the *NDC80* terminator were cloned into a *LEU2* single integration vector by Gibson Assembly (Gibson et al., 2009). LUTI promoter deletion strains were similarly constructed for each gene with the cloned upstream region only containing the sequence downstream of the LUTI TSS as determined by TL-seq. For genes in which uORF deletions were made (*APL4, HSP60, MSC6*) gBlocks with the ATG > ATC mutations were cloned by Gibson Assembly into the 3V5-tagged *LEU2* single integration vectors described above. In all strains, the WT copies of these genes remained untouched. To construct the *LexO-HSP60-ubiGFP* reporter construct, a three-piece Gibson Assembly was performed in which a *HIS3* single integration plasmid carrying *ubiGFP* was engineered to accept fragments carrying the *8xLexO-pCYC1* and the *HSP60* leader sequences. The *hsf1* mutation was generated in the above plasmid by Q5 site-directed mutagenesis (E0552s, *New England Biolabs*). All single integration plasmids were digested with PmeI before transformation and the correct integrations were confirmed by PCR. All strains and plasmids used in this study are available upon request.

pCUP1-IME1/pCUP1-IME4 sporulation

For genome-wide cell collections, cells were prepared to progress synchronously through meiosis as described in Chia and van Werven (2016). Briefly, liquid YPD (1% yeast extract, 2% peptone, 2% dextrose, tryptophan (96 mg/L), uracil (24 mg/L), and adenine (12 mg/L) cultures were started and grown for ~6 hours at 30°C until they reached an OD_{600} between 0.5 and 2.0. They were then diluted to an OD_{600} of 0.05 in reduced YPD (1% yeast extract, 2% peptone, 1% dextrose, uracil (24 mg/L), and adenine (12 mg/L)) and allowed to grow for 16-18 hours at 30°C until they reached an $OD_{600} > 6$. Cells were transferred to supplemented sporulation media or SPO (1% potassium acetate at pH 7.0 supplemented with adenine and uracil to 40 mg/L and histidine, leucine, and tryptophan to 20 mg/L, and 0.02% raffinose) with a final OD_{600} of 2.5 for 2 hours at 30°C before inducing *pCUP1-IME1* and *pCUP1-IME4* with 50 μM $CuSO_4$. In all other meiotic experiments, cells were prepared as in Carlile and Amon (2008) but with 2% potassium acetate and supplements as above. Briefly, after 24 hours of growth in YPD at RT, saturated cells ($OD_{600} > 10$) were diluted to an OD_{600} of 0.2-0.3 and inoculated in BYTA (1% yeast extract, 2% bacto tryptone, 1% potassium acetate, and 1.02% potassium phthalate) for 16-18 hours of growth at 30°C (ideally to an OD_{600} of > 5). Enough cells to give a final OD_{600} of 1.85 were transferred into SPO with 2% acetate at 30°C. After 2 hours in SPO, *IME1* and *IME4* were induced with 50 μM $CuSO_4$. In meiotic experiments with the LexA-ER-AD inducible system, cells were induced with either 2 or 5 nM of β-estradiol.

Mitotic cell collection

Exponentially growing cells from W303 background were back diluted to an OD_{600} of 0.2 and then treated with either 2 or 10 nM of β-estradiol. Cells were collected before induction as well as 1.5 and 3 hours after induction.

METHOD DETAILS

RNA extraction for TL-seq, nanopore sequencing and RNA-seq

At the indicated time points, 50 OD_{600} units of cells were collected by vacuum filtration and snap frozen in liquid nitrogen. Cells were thawed on ice and resuspended in 400 μl TES buffer (10 mM Tris pH 7.5, 10 mM EDTA, 0.5% SDS) . An equal volume of Acid Phenol:Chloroform:Isoamyl alcohol (125:24:1; pH 4.7) was added to cells, and they were incubated at 65°C for 45 minutes in a Thermomixer C (*Eppendorf*) shaking at 1400 RPM. The aqueous phase was transferred to a second tube of acid phenol. Samples were incubated at RT for 5 minutes while shaking at 1400 RPM in a Thermomixer. A final extraction with chloroform was performed. The aqueous phase was vortexed with chloroform for 30 s, separated by centrifugation, and then precipitated in isopropanol and sodium acetate for overnight at –20°C. Pellets were washed with 80% ethanol and resuspended in DEPC water for 10 min at 37°C. Total RNA was quantified using the Qubit RNA BR Assay Kit (Q10211, *ThermoFisher Scientific*).

Transcript leader sequencing (TL-seq)

The 5′ end sequencing approach was performed as in (Wu et al., 2018). At least 5 μg of mRNA was purified from total RNA using the Poly(A)Purist MAG kit (AM1922, *Ambion*). mRNAs were fragmented for 3 minutes at 70°C using a Zinc-based alkaline fragmentation reagent (AM8740, *Ambion*). RNAs were cleaned up using RNeasy MinElute Cleanup Kits (74204, *QIAGEN*) to enrich for 200-300 nucleotide fragments. These fragments were dephosphorylated with 30 units of recombinant shrimp alkaline phosphatase (M0371, *New Eengland Biolabs*) for 1 hour at 37°C with RNasin Plus (N2611, *Promega*). The RNA was extracted with Acid Phenol:-Chloroform:Isoamyl alcohol (125:24:1, pH 4.7) and precipitated in ethanol with 0.3 M sodium acetate and 1 μL linear acrylamide (AM9520, *Ambion*). RNA was then subjected to a decapping reaction with 2 units of Cap-Clip acid pyrophosphatase (C-CC15011H, *Tebu-Bio*) and with RNasin Plus. RNA was then again extracted using Acid Phenol:Chloroform:Isoamyl alcohol (125:24:1) and precipitated in ethanol. Some RNA from a premeiotic time point was set apart without the decapping reaction as a non-decapping control. Subsequently, the RNA was mixed with 10 μM of custom 5′ adaptor and the ligation reaction was done using T4 RNA ligase 1 (M0437M, *New England Biolabs*) and with RNasin Plus. The ligation reaction was cleaned up with the RNeasy MinElute Cleanup Kit (74204, *QIAGEN*) and RNAs were mixed with 2.5 μM random hexamers (N8080127, *ThermoFisher Scientific*) and RNasin Plus, denatured at 65°C for 5 minutes and cooled on ice. Reverse transcription reactions were carried out using SuperScript IV reverse transcriptase (18090010, *Invitrogen*). The RNA templates were degraded by incubating reactions with 5 units of RNase H (M0297, *New England Biolabs*) and 1.0 μL of RNase cocktail enzyme mix (AM2286, *Ambion*). DNA products were purified using 1.8x volume of HighPrep PCR beads (AC-60050, *MagBio*). Purified products were subjected to second strand synthesis using 0.3 μM of second strand biotinylated primer and the KAPA Hi-Fi hot start ready mix (KK2601, *Roche*). The second strand reaction was carried out at 95°C for 3 minutes, 98°C for 15 s, 50°C for 2 minutes, 65°C for 15 minutes and held at 4°C. Double stranded product (dsDNA) was purified with 1.8x volume HighPrep PCR beads and concentration was quantified using the Qubit dsDNA HS assay kit (Q32851, *Invitrogen*). 25 ng of dsDNA was then used as input for the KAPA Hyper Prep Kit (KK8504, *Roche*) and ligated to KAPA single indexed adapters Set B (KK8702, *Roche*). Samples were processed according to manufacturer's instructions with one exception: just prior to the library amplification step, samples were bound to MyOne Streptavidin C1 Dynabeads (65001, *ThermoFisher Scientific*) to capture biotinylated dsDNA. Library amplification over 14 PCR cycles was done on the biotinylated dsDNA fraction bound to the beads. Amplified libraries were quantified by Qubit, and adaptor-dimers were removed by electrophoresis of the libraries on Novex 6% TBE gels (EC62655BOX, *Invitrogen*) at 120 V for 1 hour, and excising the smear above ~150 bp. Gel slices containing libraries were shredded by centrifugation at 13000 *g* for 3 minutes. Gel shreds were re-suspended in 500 μL crush and soak buffer (500 mM NaCl, 1.0 mM EDTA and 0.05% v/v SDS) and incubated at 65°C for 2 hours on a thermomixer (1400 rpm for 15 s, rest for 45 s). Subsequently, the buffer was transferred into a Costar SpinX column (8161, *Corning Incorporated*) with two 1 cm glass pre-filters (1823010, *Whatman*). Columns were centrifuged at 13000 *g* for 1 minute. DNA libraries in the flowthrough were precipitated at −20°C overnight in ethanol with 0.3 M sodium acetate and 1 μL linear acrylamide (AM9520, *Ambion*). Purified libraries were further quantified and inspected on a Tapestation (*Agilent Technologies, Inc*). The libraries were sent for 100 bp SE sequencing on an Illumina HiSeq 4000 at the Vincent J. Coates Genomics Sequencing Laboratory.

polyA selection for Nanopore sequencing and RNA-seq

PolyA selection was performed on 100 μg of RNA using 150 μL of oligo-dT DynaBeads (61002, *ThermoFisher Scientific*). RNA was denatured at 80°C for 2 minutes in binding buffer (10 mM Tris-HCl pH 7.5, 0.5 M LiCl, 3.35 mM EDTA) before being placed on ice. At RT the oligo-dT beads were added to the sample and together they were incubated at room temperature for 5 minutes. Beads were washed 2x in Buffer B (10 mM Tris-HCl pH 7.5, 0.15 M LiCl, 1 mM EDTA). PolyA-selected RNA was eluted from the beads by heating at 80°C for 2 minutes in 10 mM Tris 7.0. RNA was quantified with a Qubit using the RNA HS assay kit (Q32852, *ThermoFisher Scientific*).

Nanopore sequencing

500 ng of polyA-selected RNA was used as directed in the Direct RNA Sequencing Kit (SQK-RNA001, *Oxford Nanopore Technologies*). The library was loaded onto a minION (MIN-101B, *Oxford Nanopore Technologies*) with an R9.4.1 flow cell (FLO-MIN106, *Oxford Nanopore Technologies*). MinKNOW (v1.10.23, *Oxford Nanopore Technologies*) was run without live base calling for 48 hours.

CellPress
OPEN ACCESS

Bases were called from fast5 files with the Albacore script read_fast5_basecaller.py (v2.1.10, *Oxford Nanopore Technologies*). 491,142 reads were sequenced. Reads were aligned to the genome with minimap2 (v2.9-r720; Li, 2018) using options *–ax splice –k14 –uf*. Bam files were visualized directly in IGV.

Chromatin immunoprecipitation (ChIP)

For Ume6-3V5 ChIP, 300 OD_{600} units of stationary phase cells in BYTA were collected. In histone modification experiments, 112.5 OD_{600} units of cells were collected during both premeiotic stage and meiotic prophase. In all instances, cells were fixed with 1% formaldehyde. The formaldehyde was quenched with 125 mM glycine, cells were pelleted, washed with PBS, and then lysed by Beadbeater (Mini-Beadbeater-96, *Biospec Products*) with zirconia beads for 4 × 5 minutes in FA Buffer (50 mM HEPES pH 7.5, 150 mM NaCl, 1 mM EDTA, 1% Triton, 0.1% sodium deoxycholate, 0.1% SDS supplemented with cOmplete protease inhibitor tablets (11873580001, *Roche*). Note that for the Ume6-3V5 ChIPs, due to the number of cells collected, lysates were prepared in 3 separate tubes. They were processed separately until after the IP. Lysates were collected and centrifuged for 3 minutes at 2000 g. The supernatants were transferred to fresh tubes and centrifuged for 15 minutes at 20,000 g. The supernatant was discarded, and the pellet of chromatin was resuspended in 1 mL FA Buffer. Samples were sonicated: 30 s on, 30 s off, for 5 minutes with a Bioruptor Pico (*Diagenode*) to an average fragment size of ~200 bp. The supernatant from a 1 minute 20,000 g centrifugation was carried forward to the IP.

From the isolated chromatin, 30 μL were set aside as input. For Ume6-3V5 ChIPs, to each of 3, 600 μL chromatin aliquots, 1 uL of a mouse α-V5 antibody (R960-25, *Thermo Fisher*) was added. For histone modification ChIPs, 3 μL of an antibody specific to either H3K36me3 (ab9050, *abcam*) or H3K4me2 (ab32356, abcam) was added to the chromatin. Samples were incubated for 2 hours with nutation. Protein A Dynabeads (10001D, *Invitrogen*) were blocked with 0.1% BSA in FA Buffer for at least 2 hours at 4°C. They were washed 2 x with FA Buffer, resuspended with FA Buffer to their original volume, and 10-20 μL (10 for V5 and 20 for histone modification IP) of the resuspended beads were added to each tube of chromatin. The chromatin-bead mixture was nutated at 4°C overnight. The IP was washed 6 times: 2x FA Buffer, 2x Buffer 1 (FA Buffer, 260 mM NaCl), and 2x Buffer 2 (10 mM Tris pH 8.0, 250 mM LiCl, 0.5% NP-40, 0.5% sodium deoxycholate, 1 mM EDTA). Between each wash, samples were nutated for 5 minutes at 4°C. To IP and input samples, 150 μL or 120 uL of TE (10 mM Tris pH 8.0, 1 mM EDTA) with 1% SDS was added, respectively. The precipitate was eluted from the beads by shaking at 1200 RPM in a Thermomixer (*ThermoFisher Scientific*) at 65°C. Eluates were treated with ~0.33 mg/mL RNase A (12091021, *Invitrogen*) for 30 minutes at 37°C and then with ~1.2 mg/mL Proteinase K (3115879001, *Roche*) overnight at 65°C. Samples were cleaned up with QiaQuick PCR Purification Kit (28106, *QIAGEN*) and eluted in EB. DNA was quantified by Qubit with the dsDNA HS Assay Kit (Q32854, *Invitrogen*). Libraries were prepared as instructed by the ThruPLEX DNA-seq Kit (R400427, *Takara*). For input and H3K36me3 IP samples, 15 ng of starting material was used with 7 rounds of PCR. For all other IP samples, 0.5-1 ng of starting material was used with 11 rounds of PCR. AMPure XP beads (A63881, *Beckman Coulter*) were used to select fragments between 200-500 bp. Samples were submitted for 50 bp SE sequencing by the Vincent J. Coates Genomics Sequencing Laboratory with a HiSeq4000.

RNA-seq

The RNA-seq libraries were prepared using the NEXTflex™ Rapid Directional mRNA-Seq Kit (NOVA-5138, *Bioo Scientific*) according to manufacturer's instructions. 100 ng of polyA selected mRNA was used for all libraries. Libraries were quantified using the Agilent 4200 TapeStation (*Agilent Technologies, Inc.).* AMPure XP beads (A63881, *Beckman Coulter*) were used to select fragments between 200-500 bp. Samples were submitted for 150 bp SE sequencing by the Vincent J. Coates Genomics Sequencing Laboratory with a HiSeq4000.

Immunoblotting

Immunoblotting was performed as previously described in Chen et al. (2017). To track tagged proteins of interest, membranes were incubated with either a mouse α-V5 antibody (R960-25, *Thermo Fisher*) or a mouse α-GFP antibody (632381, *Takara*) to a dilution of 1:2000 in Odyssey Blocking Buffer (PBS) (*LI-COR Biosciences*) with 0.01% Tween. Both V5 and GFP immunoblots were also incubated with a rabbit α-hexokinase (α-Hxk2) antibody (H2035, *US Biological*) diluted between 1:15,000-1:20,000. Secondary antibodies included a α-mouse antibody conjugated to IRDye 800CW (926-32212, *LI-COR Biosciences*) and a α-rabbit antibody conjugated to IRDye 680RD (926-68071, *LI-COR Biosciences*). They were each diluted to 1:15,000 in Odyssey Blocking Buffer (PBS) with 0.01% Tween. An Odyssey system (*LI-COR Biosciences*) was used to image all blots, and Image Studio Lite (*LI-COR Biosciences*) was used for all quantification.

RNA extraction for blotting and RT-qPCR

At the indicated time points, between 4 and 5 OD_{600} units of cells were collected by centrifugation and snap frozen in liquid nitrogen. Cells were thawed on ice and resuspended in TES (10 mM Tris pH 7.5, 10 mM EDTA, 0.5% SDS). An equal volume of Acid Phenol:-Chloroform:Isoamyl alcohol (125:24:1; pH 4.7) was added to cells, and they were incubated at 65°C for 45 minutes in a Thermomixer C (*Eppendorf*) shaking at 1400 RPM. The aqueous phase was transferred to a second tube with chloroform. The aqueous phase was

vortexed with the chloroform for 30 s, separated by centrifugation, and then precipitated in isopropanol and sodium acetate overnight at –20°C. Pellets were washed with 80% ethanol and resuspended in DEPC water for 10 min at 37°C. Total RNA was quantified by Nanodrop.

RNA blotting

RNA blot analysis protocol was performed as described previously (Koster et al., 2014) with minor modifications. 8 μg of total RNA was denatured in a glyoxal/DMSO mix (1M deionized glyoxal, 50% v/v DMSO, 10 mM sodium phosphate (NaPi) buffer pH 6.5–6.8) at 70°C for 10 minutes. Denatured samples were mixed with loading buffer (10% v/v glycerol, 2 mM NaPi buffer pH 6.5–6.8, 0.4% w/v bromophenol blue) and separated on an agarose gel (1.1% w/v agarose, 0.01 M NaPi buffer) for 3 hr at 100 V. The gels were then soaked for 25 minutes in denaturation buffer (0.05 N NaOH, 0.15 M NaCl) followed by 20 minutes in neutralization buffer (0.1 M Tris-HCl pH 7.5, 0.15 M NaCl). RNA was transferred to nitrocellulose membrane for 1 hour via vacuum transfer as described in Stratagene's Membranes Instruction Manual (*Stratagene*) and crosslinked using a Stratalinker UV Crosslinker (*Stratagene*). rRNA bands were visualized using methylene blue staining. The membranes were blocked in ULTRAhyb Ultrasensitive Hybridization Buffer (AM8669, *ThermoFisher Scientific)* for 3 hours before overnight hybridization. Membranes were washed twice in Low Stringency Buffer (2x SSC, 0.1% SDS) and three times in High Stringency Buffer (0.1X SSC, 0.1% SDS). All hybridization and wash steps were done at 42°C. Radioactive probes were synthesized using a Prime-It II Random Primer Labeling Kit (300385, *Agilent Technologies, Inc*).

Micrococcal nuclease sequencing (MNase-seq)

The protocol was adapted from Basic Protocol 1 in Rodriguez et al. (2014) with the following changes. In premeiotic stage and meiotic prophase, 112.5 OD_{600} units of cells were fixed in 1% formaldehyde with light shaking at RT for 15 minutes. Crosslinking was quenched by 125 mM of glycine for 5 minutes at RT. Cells were pelleted and washed twice with ice cold milliQ water. Cells were spheroplasted in 20 mL of Spheroplast Solution (1 M Sorbitol, 50 mM Tris pH 7.5, 10 mM β-ME) with 100 μL of 10 mg/mL zymolase until they appeared non-refractive and shadow-like after ~20-30 minutes. Spheroplasted cells were resuspended in 2 mL MNase Digestion Buffer (1 M Sorbitol, 50 mM NaCl, 10 mM Tris pH 7.5, 5 mM $MgCl_2$, 1 mM $CaCl_2$, 0.075% NP-40, 0.5 mM spermidine, 1 mM β-ME) if collected during premeiotic stage and 4 mL if collected during meiotic prophase, after completion of S-phase. Digestions were performed with 600 uL of spheroplasts, 30 units of Exonuclease III (M0206S, *New England Biolabs*), and either 10, 20, or 40 units of MNase (LS004797, *Worthington*). Crosslinks were reversed, protein was degraded by Proteinase K (3115879001, *Roche*), and a phenol/chloroform/isoamyl alcohol DNA extraction, ethanol precipitation, RNase A (12091021, *Invitrogen*) treatment, and phosphatase treatment were performed as described previously (Rodriguez et al., 2014). Size selection was performed by running samples on a 1.8% LMT agarose gel at 80 V for 40 minutes at room temperature and gel extracting the mononucleosome band with a Monarch Gel Extraction Kit (T1020S, *New England Biolabs*). Note that of the samples digested with 10, 20, and 40 units of MNase, only the samples with a ratio of mononucleosomes to dinucleosomes closest to 80/20 were size selected and carried forward for library preparation. Gel extracted samples were quantified by Qubit with the dsDNA HS Assay Kit (Q32854, *Invitrogen*). Libraries were prepared with 50 ng starting material as instructed by the ThruPLEX DNA-seq kit (R400427, *Takara*). Amplification was performed with 5 rounds of PCR. AMPure XP beads (A63881, *Beckman Coulter*) were used to select fragments between 150-500 bp. Samples were submitted for 100 bp PE sequencing to the Vincent J. Coates Genomics Sequencing Laboratory with a HiSeq4000.

Reverse transcription-quantitative polymerase chain reaction (RT-qPCR)

To 5 μg of isolated total RNA in 1x DNase I reaction buffer, 1 unit of DNase I was added. The reaction was incubated at 37°C for 30 minutes. The DNase-treated RNA was extracted by Acid Phenol:Chloroform:Isoamyl alcohol (125:24:1; pH 4.7) and precipitated with isopropanol and sodium acetate overnight. The RNA was washed in 80% ethanol and resuspended in milliQ water. cDNA was reverse transcribed following the Superscript III kit (18080044, *ThermoFisher Scientific*). Quantification was performed with Absolute Blue qPCR Mix (AB4162B, *ThermoFisher Scientific*). The primers used to quantify $ubiGFP^{LUTI}$ included a forward primer which annealed immediately upstream of the $HSP60^{PROX}$ TSS and a reverse primer which annealed to the first bases of ubiquitin. Meiotic signals were normalized to *PFY1* and mitotic signals were normalized to *ACT1*. Oligonucleotides are in Table S7.

QUANTIFICATION AND STATISTICAL ANALYSIS

TL-seq analysis

From the sequencing reads, the 3′ Illumina adaptor (AGATCGGAAGAGC) was trimmed using cutadapt with the–*minimum-length* option set to 20 bp (v2.3; Martin, 2011). From the 3′ trimmed output, the 5′ Illumina adaptor (CACTCTGAGCAATACC) was trimmed from reads by cutadapt. To select for reads with the most 5′ end of a transcript, only reads in which the 5′ adaptor was recognized and then trimmed were carried forward. Reads were aligned by STAR (v2.5.3a; Dobin et al., 2013) using indices generated from an SK1 genome assembled by combined PacBio and Illumina sequencing (Yue et al., 2017). A custom SK1 genome was forged with BSgenome (v1.50.0; Pagè, 2018) using the above assembly (Yue et al., 2017). Bam files were imported into CAGEr and the CAGEr pipeline was applied to define TSSs and quantify transcript abundances as follows (v1.24.0; Haberle et al., 2015). Reads at TSSs were

counted (*getCTSS*) and normalized by *"simpleTpm"* (*normalizeTagCount*). An initial clustering was performed (*clusterCTSS* with *threshold = 2*, *thresholdIsTpm = TRUE*, *method = "distclu"*, *maxDist = 5*, *removeSingletons = TRUE*, and *keepSingletonsAbove = 3*), and the output was aggregated into larger clusters representative of all the activity expected from a single promoter (*aggregateTagClusters* with *tpmThreshold = 1* and *maxDist = 50*). Clustered TSSs were exported as bedGraph files for visualization in IGV (*exportCTSStoBedGraph* with *values = "normalized"*). Cluster counts were exported to DESeq2 by time point (*concensusClustersDESeq2*), and fold-changes were calculated by DESeq2 with default settings including a FDR of 0.1. Output from this clustering was used to define TSSs coordinates of 5′-extended transcripts in the pipeline below. A secondary and more permissive clustering (*threshold = 1, thresholdIsTpm = TRUE, method = "distclu," maxDist = 5, removeSingletons = FALSE*) was performed after LUTIs were defined. Output from the secondary clustering was used for quantification and in all presented TL-seq scatterplots. For all scatterplots in Figure 2, Figure S3, Figure 4, and Figure S7, the Spearman's correlation coefficient was calculated and is displayed on each plot. All TL-seq comparisons to RNA-seq were performed in duplicate. The TL-seq used to define LUTI promoters was performed in triplicate. In Figure 4D a Wilcoxon signed-rank test was performed on data that was collected in duplicate. The p value is included in the figure legend.

Pipeline for 5′-extended transcript discovery

Using the output from DESeq2 after CAGEr, TSS clusters were filtered for coordinates in which the mean over both time points was > 2 transcripts per million and the log2 fold-change as cells entered meiotic prophase compared to premeiotic was > 2. After applying these filters, the coordinates for each peak were manually inputted into IGV. The TL-seq peak was compared to nanopore sequencing reads from a sample taken during meiotic prophase (4 hours). If at least one Nanopore read extended from a region near the TSS coordinates and continued uninterrupted across the entirety of a neighboring CDS, the coordinates were marked for continued investigation. Purely intergenic and either 5′ or 3′ truncated transcripts were removed in this way. From the remaining subset of peaks, a 5′-extension was only called if a second promoter, downstream, but on the same strand, was closer to the CDS. Through this criterion, canonical meiosis-specific genes were eliminated from the analysis. It resulted in 74 candidate LUTIs with 5′extensions. For downstream analyses, the single most dominant bp in each TSS cluster was determined by a custom python script.

Motif discovery

Meme was applied to the sequences 300 bp up and 300 bp downstream of each LUTI TSS with options *–w 10 –dna –revcomp.*(Bailey and Elkan, 1994). A motif was considered significant in an individual sequence if it had a combined match p value < 0.05.

Ume6 ChIP-seq analysis

Reads were aligned to the SK1 genome with bowtie2 (v2.3.4.3; Langmead and Salzberg, 2012; Langmead et al., 2019). Using randsample from macs2, all libraries were down-sampled to 2 million reads (v2.1.1.20160309; Zhang et al., 2008). Macs2 callpeak was used to call peaks in IP samples over input samples with options *–B –q 0.001–keep-dup "all"–call-summits –nomodel –extsize 147*. Bigwig files for viewing in IGV were generated by macs2 bdgcmp with option *–m FE* followed by bedGraphToBigWig (v4; Kent et al., 2010). Heatmaps and metagene plots centered around TSSs as defined by TL-seq were constructed with deeptools2 (v3.0.1; Ramírez et al., 2016). A 5′-extended or canonical target promoter was considered to be enriched by Ume6 if, in at least 2 of 3 ChIP replicates, a peak was called (log2 fold-change > 2 over input) within 300 bp of the transcript's TSS.

Conservation of URS1 binding sites

The 5′-extended and canonical Ume6 targets enriched with both Ume6 and a URS1 binding site in their promoters were analyzed for degree of conservation. Meme was run on the sequences ± 300 bp from their TSSs with options *–w 10 –dna –revcomp* was run to identify the location of the URS1 binding sites with regard to the TSS. Using a custom python script, the midpoint of each URS1 binding motif was determined relative to the CDS associated with either the LUTI or canonical target. Subsequently, the chromosome locations of the URS1 midpoint in the sacCer3 S288C reference genome were found. To assess conservation of the regions around URS1 motifs at 5′-extended and canonical targets, phastCons (Siepel et al., 2005) was performed with options *–target-coverage 0.025 -expected-length 12 –rho 0.4*. The tree phylogeny model and the genome alignments of *S. cerevisiae, S. paradoxus, S. mikatae, S. kudriavzevii, S. bayanus, S. castelli, and S. kluyveri* were from Siepel et al. (2005). However, the alignments of *S. castelli* and *S. kluyveri* were excluded in all analyses here because these yeast species have lost the *IME1* and *UME6* genes. Metagene plots and heatmaps of conservation were generated with deeptools2 (v3.0.1; Ramírez et al., 2016)

Gene set enrichment analysis (GSEA)

Normalized counts for each gene were calculated by DESeq2 using default options (v1.22.2; Love et al., 2014). The *Saccharomyces cerevisiae* collection of gene sets (updated from SGD on 2020-12-28) was downloaded from the Gene Ontology Consortium (Ashburner et al., 2000; Carbon et al., 2021). The collection was supplemented with a gene set including the 41 genes identified in Figure 2A of Brar et al. (2012) as being expressed during recombination/synaptonemal complex formation. GSEA was performed using the desktop app version 4.1.0 with the Collapse/Remap to gene symbols setting set to "No_Collapse" and the permutation type defined as gene_set. All other parameters were default settings.

uORF analysis

ATGs were counted and the codon frequency was determined with a custom python script. For genes with LUTIs, the counts and codon frequencies were determined for the region between the PROX TSS and the LUTI TSS. For all other genes, sequences from the 500 bp upstream of the TSS were used.

LUTIs with > 4 uORFs were analyzed to determine which of the uORFs were translated. Footprints were quantified for the first 6 codons of each uORF using the tools in Brar et al. (2012) and Ingolia et al. (2009). The ribosome footprinting data were taken from the 3 h time point in Cheng et al. (2018). Any uORFs with at least 4 footprint reads found across the first 6 codons of the gene were considered to be translated.

Chromatin modification analysis

Reads were aligned to the SK1 PacBio genome assembly with bowtie2 (v2.3.4.3; Langmead and Salzberg, 2012). Macs2 callpeak was used to call peaks in IP samples over input samples with options *–B –q 0.01 –nomodel –extsize 147*. Bigwig files for viewing in IGV and for further quantification were generated by macs2 bdgcmp with option *–m FE* followed by bedGraphToBigWig (v4; Kent et al., 2010; Zhang et al., 2008). To quantify the change in H3K36me3 and H3K4me2 enrichment over the promoters of PROX transcripts, fold enrichment scores were extracted from regions 50 bp upstream and 500 bp downstream of the PROX TSS with bedtools (Quinlan and Hall, 2010). With custom python scripts, the scores from each bp of the upstream regions and each bp of the downstream regions were summed for each gene. The ratio of the upstream and the downstream region enrichments were quantified and the change in the score from premeiotic stage to meiotic prophase was determined. Ultimately, the mean of the FC was calculated from samples in triplicate. Heatmaps and metagene plots were prepared with deeptools2 (Ramírez et al., 2016).

RNA-seq analysis

Quantification of RNA as transcripts per million was done using salmon in the mapping-based mode with mapping validation (v0.13.1; Patro et al., 2017). FC quantification was performed by DESeq2 using default options (v1.22.2; Love et al., 2014). Scatterplots were generated with matplotlib (Hunter, 2007). For all scatterplots in Figures 2 and S3 with RNA-seq data, the spearman correlation coefficient was calculated and is displayed on the plot. Heatmaps were generated with pheatmap.

MNase-seq analysis

Reads were aligned to the SK1 genome with bowtie2 (v2.3.4.3; Langmead and Salzberg, 2012). To select for only fragments between 130 and 170 bps, alignmentSieve from deeptools2 was performed (Ramírez et al., 2016). BigWig files were generated by bamCoverage with options --*MNase –bs 1–normalizeUsing CPM* (Ramírez et al., 2016). DANPOS (v2.2.2) was run to determine various aspects of nucleosome location and occupancy and fuzziness (Chen et al., 2013). A custom python script was used to assign the locations of +1 and –1 nucleosome with respect to PROX TSSs.

Clustered heatmap

Spearman’s correlation coefficients were calculated between all pairs of features in 6A. The coefficients are displayed on the scatterplots in Figure S7. P values of significant features are indicated in the text.

Molecular Cell, Volume *81*

Supplemental information

Integrated genomic analysis reveals key features of long undecoded transcript isoform-based gene repression

Amy Tresenrider, Kaitlin Morse, Victoria Jorgensen, Minghao Chia, Hanna Liao, Folkert Jacobus van Werven, and Elçin Ünal

Supplemental Figure Legends:

Figure S1. TL-seq and direct RNA sequencing for a LUTI gene and a meiosis-specific gene, Related to Figure 1

A. Genome browser views of TL-seq and direct RNA sequencing using Nanopore at the *NDC80* locus. The TL-seq and Direct RNA sequencing were performed in cells with the *pCUP-IME1/pCUP-IME4* meiotic induction system (*UB14584*). The premeiotic time point was taken after 2 hours in SPO, and the meiotic prophase time point was taken after 4 hours in SPO, which corresponds to 2 hours after 50 µM $CuSO_4$ addition. **B.** As in **(A)** but for *SPO11*.

Figure S2. Ume6 enrichment for the genes without 5' extensions and RNA-seq browser from *UME6* and *ume6* T99N strains, Related to Figure 1

A. Heatmap of Ume6 enrichment over input for all of the genes without 5'-extensions (n=5298). A representative image from one of three replicates is shown. **B-C.** Genome browser views of RNA-seq at the **(B)** *NDC80* and the (**C**) *APL4 locus.* RNA-seq was performed on strains expressing either WT Ume6 (*UB20649*) or Ume6 with a T99N mutation (*UB22629*). The strains also harbor the *pCUP1-IME1/pCUP1-IME4* meiotic induction system and were induced with 50 µM $CuSO_4$ at 2 hours in sporulation medium (SPO). Cells were collected after 2 or 4 hours in SPO.

Figure S3. Contribution of LUTI and PROX isoforms to total RNA abundance and assessment of RNA-seq similarity between this study and Cheng et al. 2018, Related to Figure 2

A. Scatterplots comparing RNA-seq and TL-seq. Cells harboring the *pCUP1-IME1/pCUP1-IME4* meiotic induction system and were induced with 50 µM $CuSO_4$ at 2 hours in sporulation medium (SPO). Analysis was performed with data from cells collected during meiotic prophase (4 hours in SPO). In all plots the teal data points display RNA-seq vs TL-seq transcripts per million for genes without 5'-extensions. All orange points indicate loci with 5'-extended transcripts. TL-seq values for genes with 5'-extended transcripts were calculated either from PROX-specific TPM (Left), LUTI-specific TPM (Middle), or the addition of both PROX- and LUTI-specific TPM (Right). The Spearman's rank correlation coefficient (ρ) was calculated and is displayed on the upper left corner for both teal and orange points separately. **B.** Scatterplots comparing RNA-seq performed in this study to RNA-seq performed in Cheng et al. 2018. In this study, cells with the *pCUP1-IME1/pCUP1-IME4* meiotic induction system (*UB14584*) were collected for RNA-seq (in duplicate) after 4 hours in sporulation medium (SPO), which corresponds to 2 hours after induction of meiosis by 50 µM $CuSO_4$. In Cheng et al., a prototrophic strain was used. Cells were collected after 1.5 (left), 3 (middle) or 4.5 (right) hours in SPO. The Spearman's rank correlation coefficient (ρ) was calculated and is displayed on the upper left corner. **C.** GSEA enrichment plot for the SC/Recombination gene set defined in Brar et al. (2012). Analysis was performed with RNA-seq (duplicates) collected from cells harboring the*pCUP1-IME1/pCUP1-IME4*

meiotic induction system (*UB14584*). Enrichment was determined by comparing gene expression at 4 hours in SPO (meiotic prophase) to 2 hours (premeiotic).

Figure S4. The effect of uORF deletion or *upf1Δ* on LUTI abundance and protein level, Related to Figure 3

A. Cells with either wild-type uORFs or uORFs with ATGs mutated to ATC and a 3V5 tagged *APL4* (*WT: UB18539, uORF*Δ: *UB26122*), *HSP60* (*WT: UB18335, uORFΔ:UB26123*), or *MSC6* (*WT: UB18238, uORFΔ:UB26124*) were induced to undergo meiosis with 50 µM $CuSO_4$ after 2 hours in SPO using the *pCUP1-IME1/pCUP1-IME4* meiotic induction system. Immunoblot was performed against the 3V5 epitope and Hxk2 as a loading control. One of two replicates is displayed. **B-M.** Wild-type or *upf1Δ* strains harboring 3V5 tagged *APL4* (*WT: UB18539, upf1*Δ: *UB26707*), *HSP60* (*WT: UB18335, upf1Δ:UB26705*), or *MSC6* (*WT: UB18238, uORFΔ:UB26708*) were induced to undergo meiosis with 50 µM $CuSO_4$ after 2 hours in SPO using the *pCUP1-IME1/pCUP1-IME4* meiotic induction system. (**B-D**) RNA blots whereby transcripts were detected using a probe directed against the 3V5 epitope and its linker sequence. rRNA was stained by methylene blue as a loading control. (**E-G**) qPCR quantification of LUTI transcripts using the matched RNA from **B-D.** Quantification was performed in reference to the levels of the meiotic housekeeping gene *PFY1*. **(H-J)** Immunoblot with α-3V5 and α-Hxk2 antibodies. (**K-M**) Quantification of immunoblots in **H-J.** Abundance of the 3V5 tagged protein was quantified by first normalizing to the Hxk2 loading control and then to the first time point (0 h).

Figure S5. Single gene analysis of LUTI deletion, Related to Figure 4

A-B. LUTI deletion analysis of additional genes. Cells with the *pCUP1-IME1/pCUP1-IME4* meiotic induction system were induced to enter meiosis with 50 µM $CuSO_4$ after 2 hours in SPO. **A.** Experiments were performed with a *MSC6-3V5* tagged strain harboring either wild type (*UB18238*) or LUTI deletion (*UB18190*). RNA blots and Immunoblots were performed on samples collected between 0-6 hours in SPO. RNA blots were performed with a probe specific for 3V5 and its linker. Methylene blue detection of rRNA bands was the loading control. Immunoblots were performed with a α-V5 antibody to recognize the 3V5-tagged proteins. Hxk2 was used as a loading control. The blots represent one of two replicates. Quantification of the immunoblot is displayed on the right. 3V5 signal was quantified relative to Hxk2 and then relative to 0 hr. Graphs represent one of two replicates **B.** Same as A, but for *APL4-3V5* tagged strains with either wild type (*UB18539*) or LUTI deletion (*UB18181*).

Figure S6. Alterations to chromatin over the PROX promoter upon LUTI expression, Related to Figure 5

A. Metagene analysis of H3K36me3 ChIP-seq for LUTI and non-LUTI genes in both the premeiotic and meiotic prophase stages. The ChIP was performed in cells with the *pCUP1-IME1/pCUP1-IME4* meiotic induction system *(UB14584).* The premeiotic time point was taken after 2 hours in SPO, and the meiotic prophase time point was taken

after 4 hours in SPO, which corresponds to 2 hours after 50 µM $CuSO_4$ addition. The images are from one of three replicates. **B.** Same as (**A**) but for H3K4me2. **C.** Heatmap of H3K36me3 ChIP-seq in premeiotic and meiotic prophase. The ChIP was performed in cells with the *pCUP1-IME1/pCUP1-IME4* meiotic induction system (*UB14584*). The premeiotic time point was taken after 2 hours in SPO, and the early meiotic time point was taken after 4 hours in SPO, which corresponds to 2 hours after 50 µM $CuSO_4$ addition. The plot is centered around the PROX TSS. Top: LUTIs, bottom: canonical transcripts. The images are from of one of three replicates. **D.** Same as **(C)** but for H3K4me2. **E-F.** Genome browser views of MNase-seq and TL-seq. The TL-seq and MNase DNA digestion was performed in cells with the *pCUP-IME1/pCUP-IME4* meiotic induction system (*UB14584*). The premeiotic time point was taken after 2 hours in SPO, and the meiotic prophase time point was taken after 4 hours in SPO, which corresponds to 2 hours after 50 µM $CuSO_4$ addition. Three candidate genes, *RPO41* **(E)**, *APL4* **(F)** and *SWI4* **(G)**, all of which are robustly transcriptionally repressed are shown. The green box denotes the approximate location of the PROX promoter.

Figure S7. Reduction in PROX transcript abundance shows significant correlation with a select number of criteria, Related to Figure 6

Scatterplots of the relationships tested in Figure **6A**. Spearman's correlation coefficients (ρ) are displayed on the upper left corner.

Supplemental Table Legends:

Table S5. Translational efficiency (TE) fold-change between meiotic prophase and premeiotic stage for LUTIs with < 4 ATG codons in the region between the LUTI and the PROX TSSs, Related to Figure 3

Table S7. Primers used in this study, Related to STAR Methods

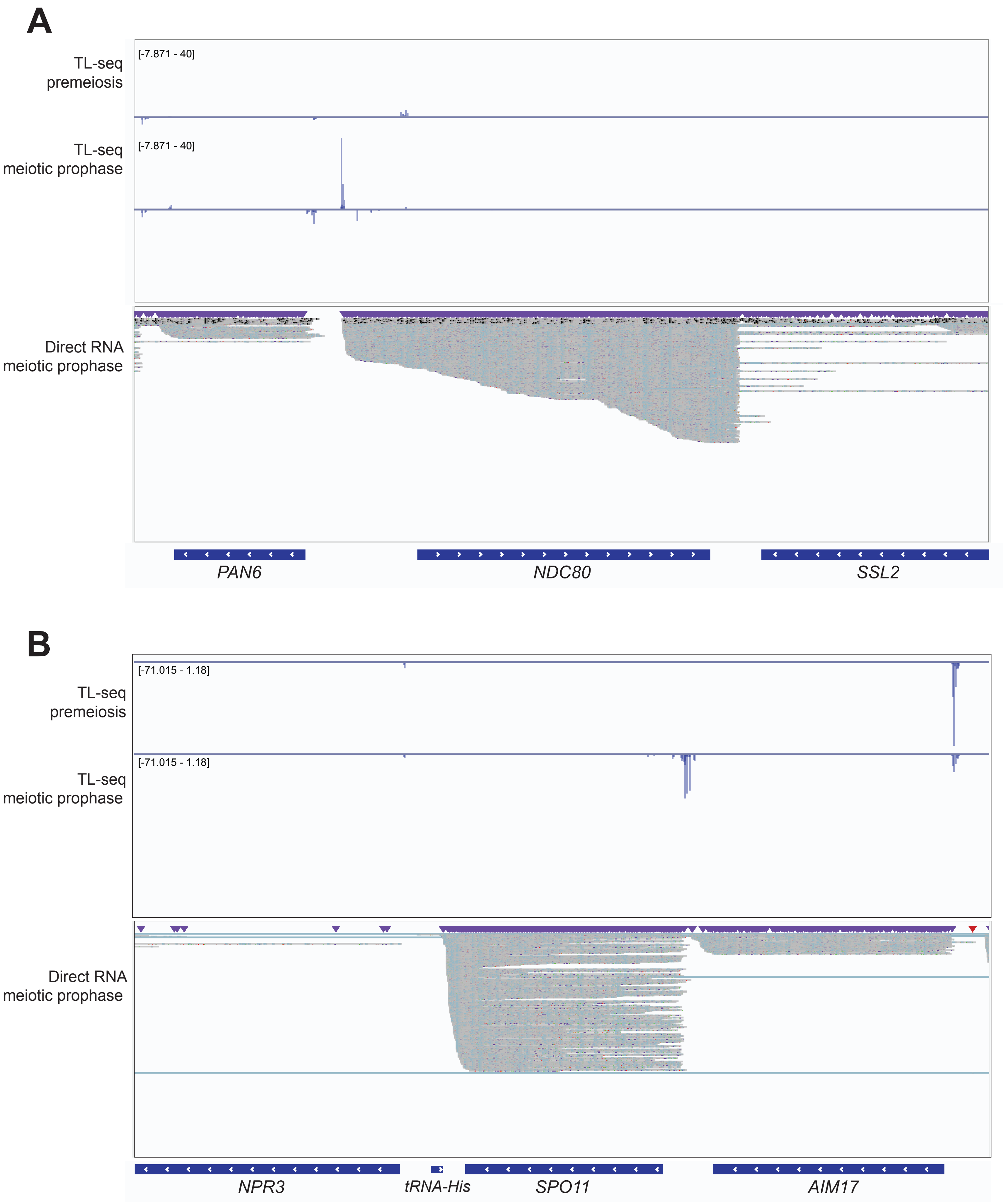

Figure S1, Tresenrider et al.

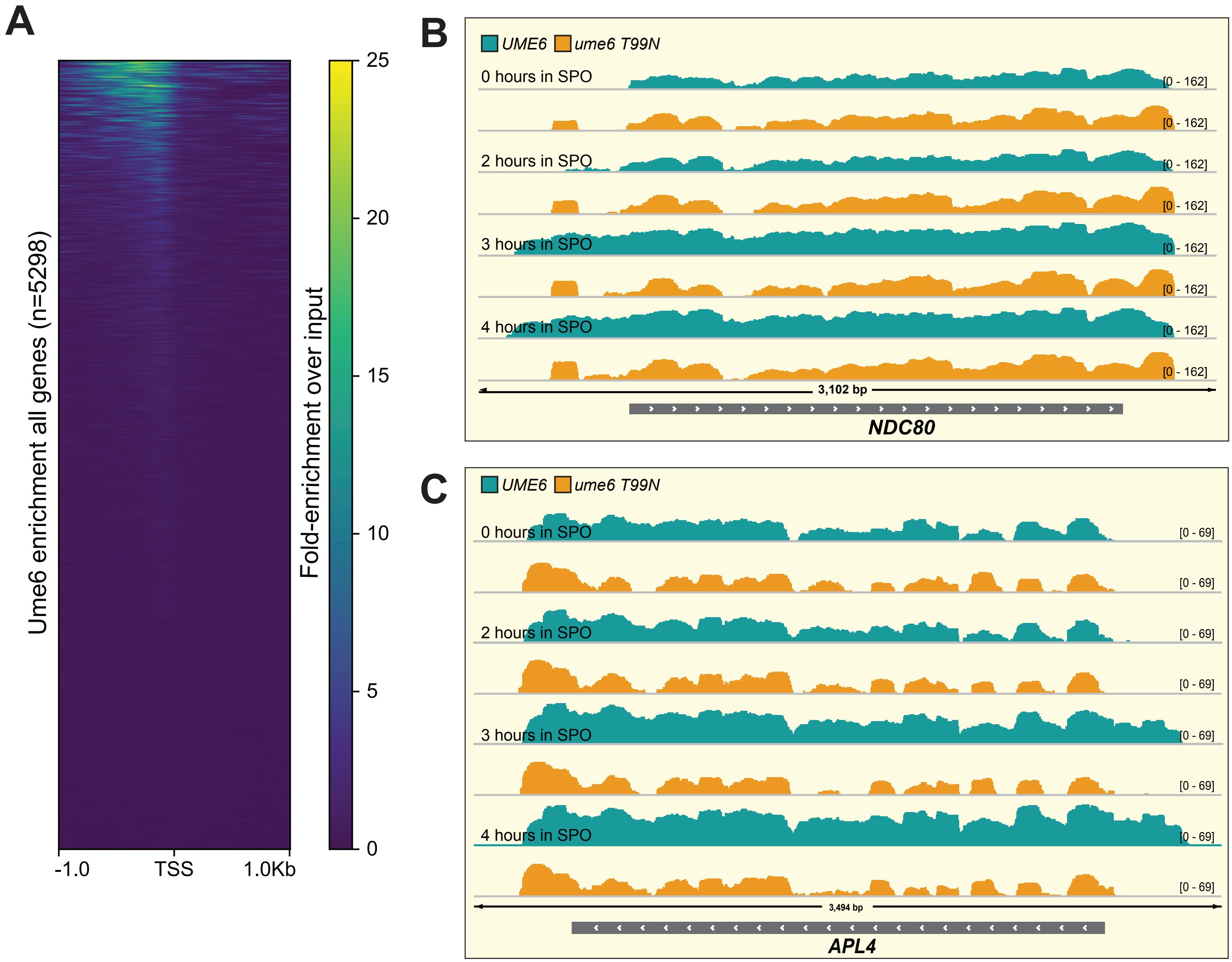

Figure S2, Tresenrider et al.

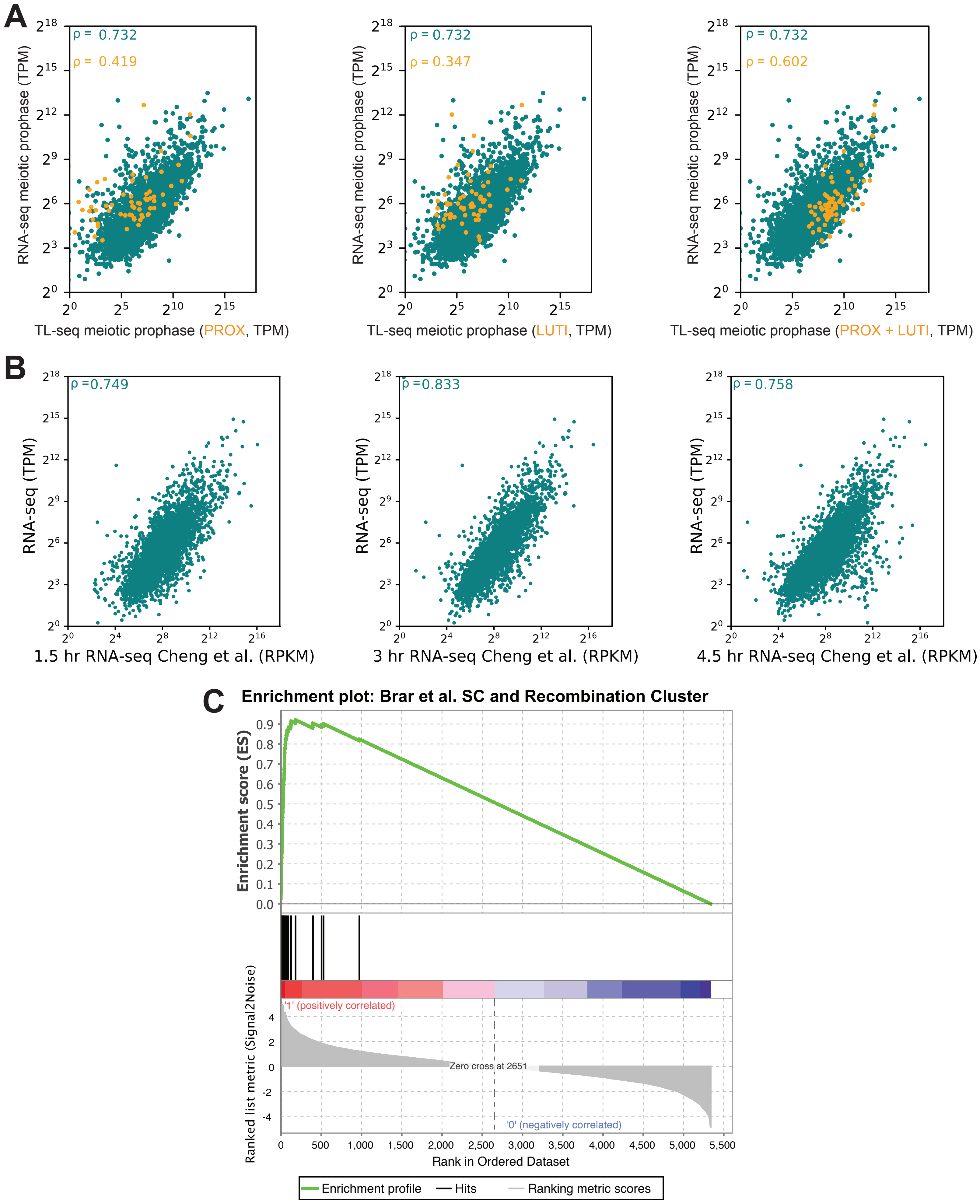

Figure S3, Tresenrider et al.

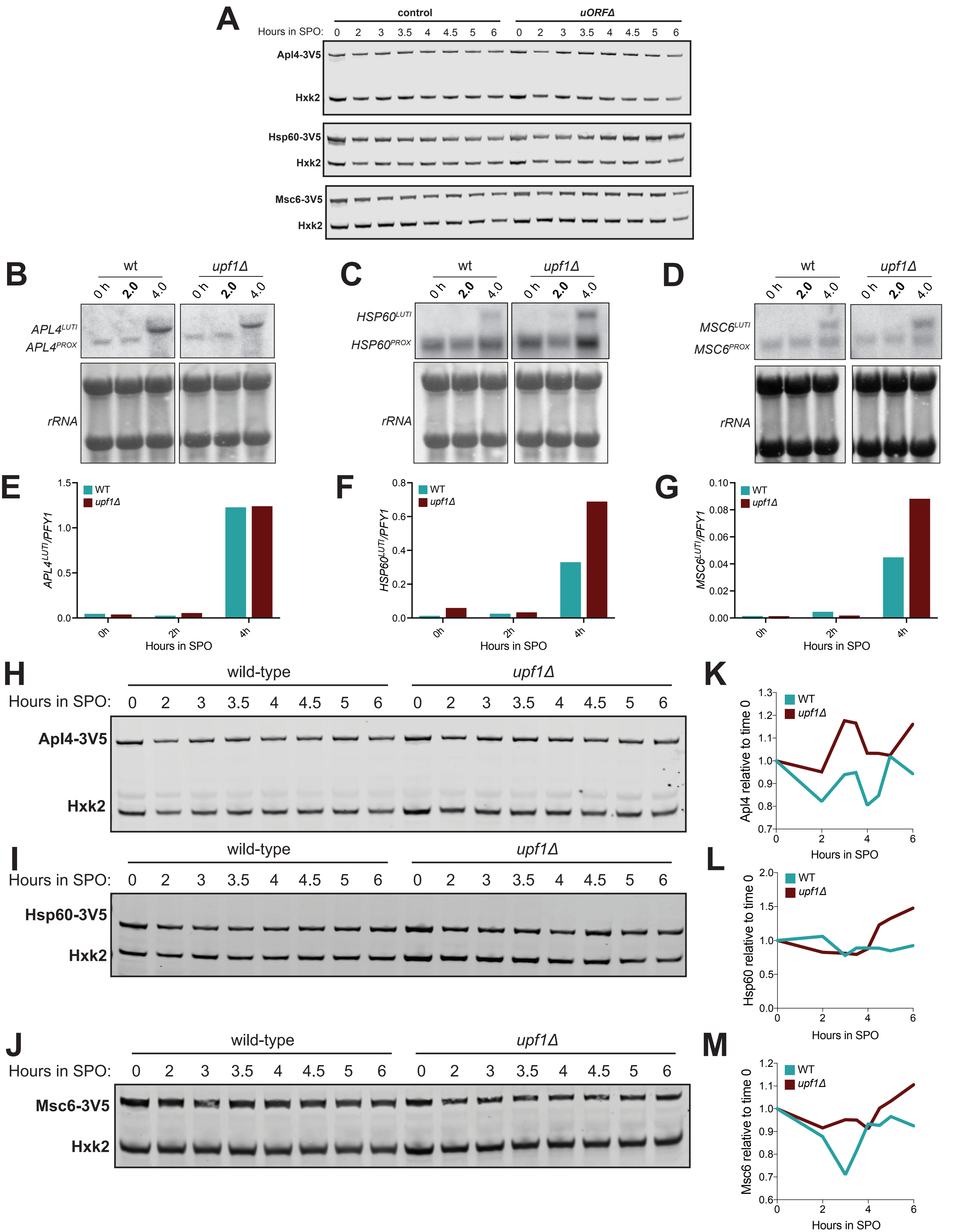

Figure S4, Tresenrider et al.

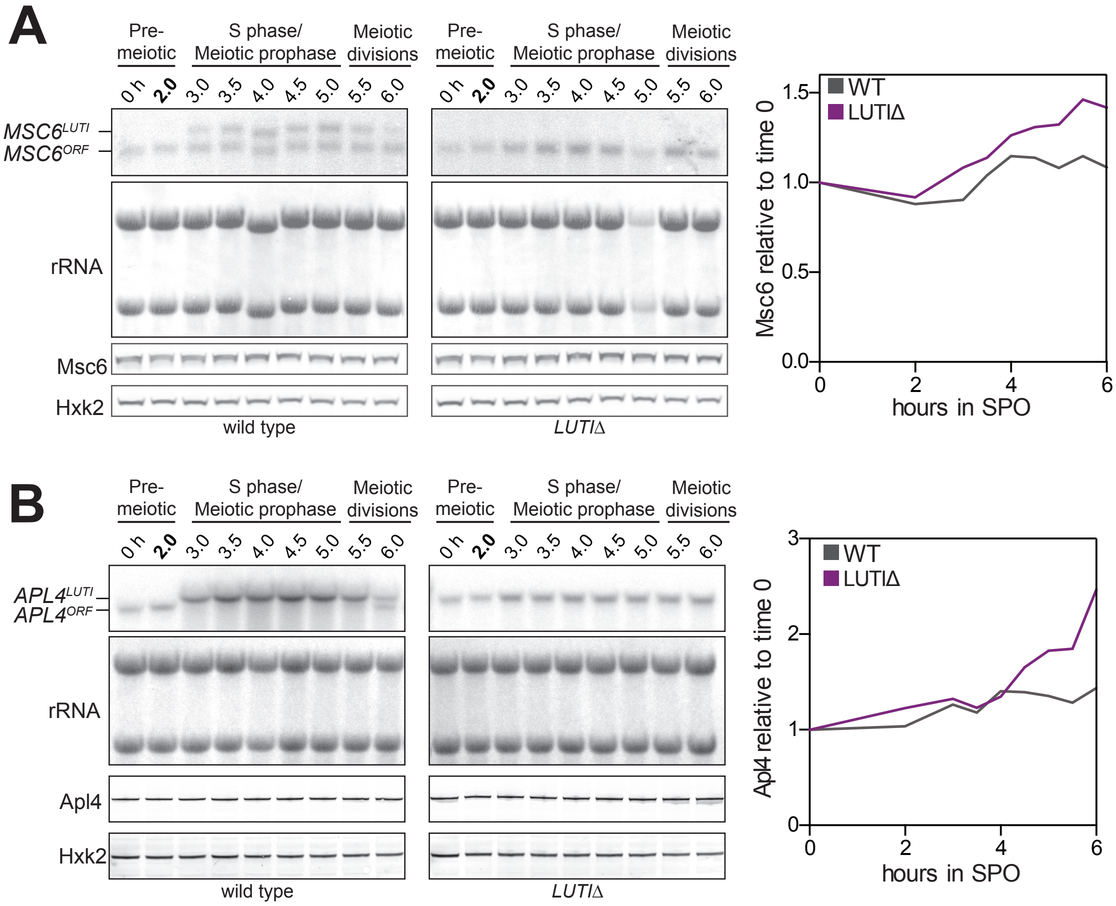

Figure S5, Tresenrider et al.

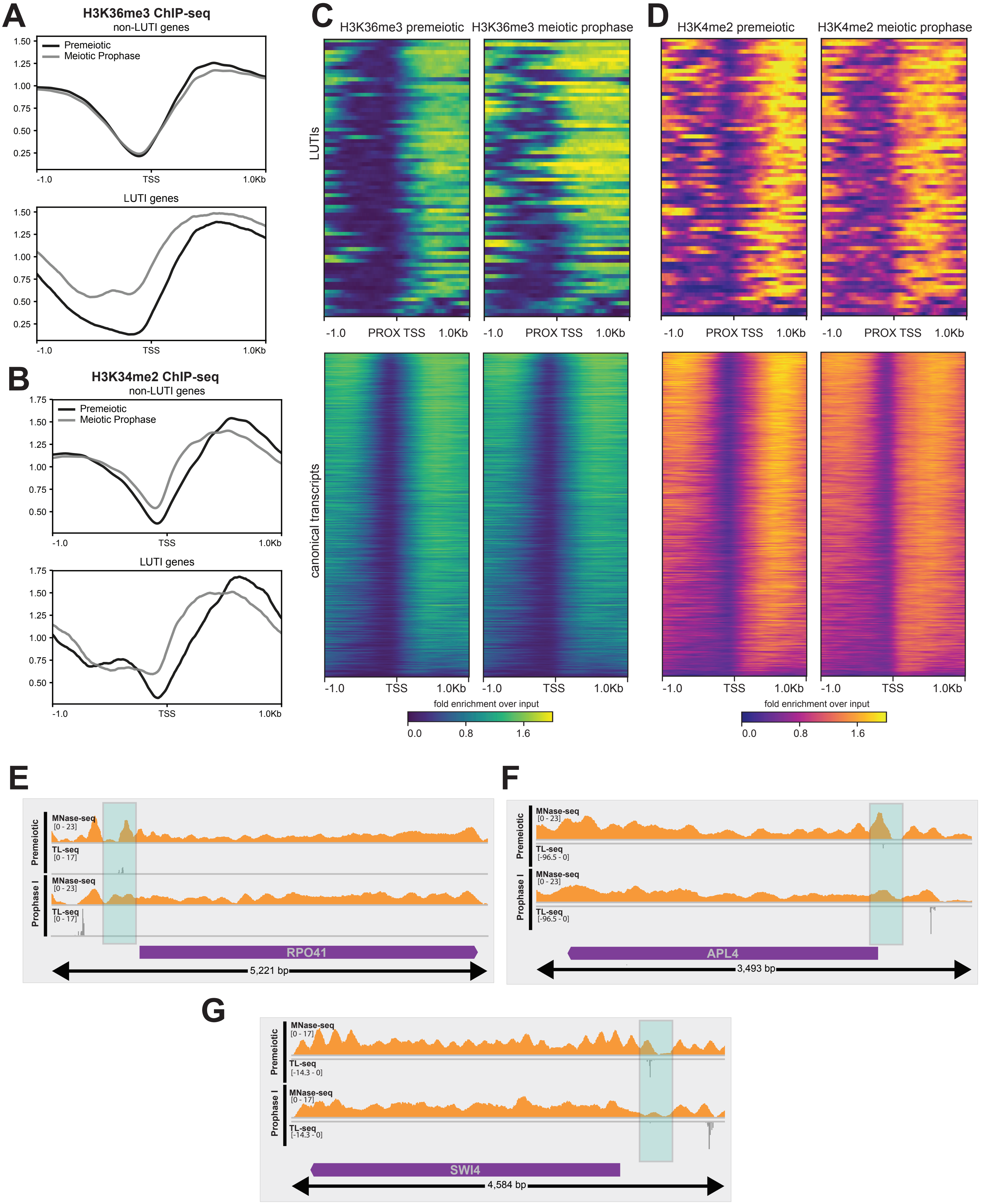

Figure S6, Tresenrider et al.

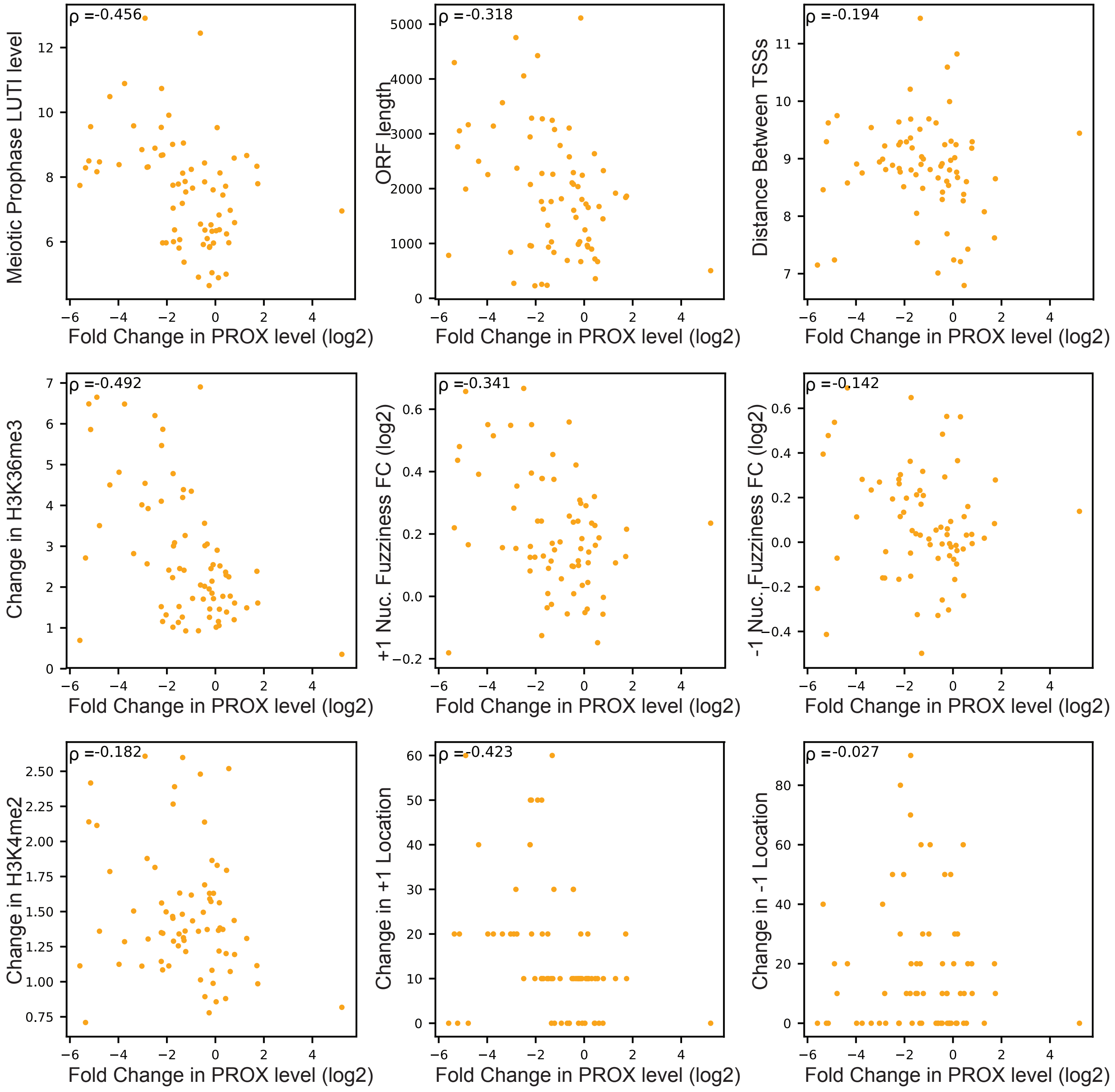

Figure S7, Tresenrider et al.

Table S5. LUTI candidates with fewer than 4 uORFs

Gene	uORF number	TE fold-change
PLM2	0	4.894
YCL057C-A	0	3.411
ELO1	1	1.078
COX16	1	0.995
ULP2	1	0.057
MNE1	1	0.005
ITR2	2	1.906
YNL305C	2	0.193
VPS30	3	0.236
APL4	3	0.193

Table S7. Primers used in this study.

**Primer Name**	**Oligonucleotide sequence from 5’ to 3’**
3V5_probe_F	CTAGTGGATCCAGGTAAACCTAT
3V5_probe_R	TAATACGACTCACTATAGGCCAGTCCT AATAGAGGATTAGG
ACT1_F	GTACCACCATGTTCCCAGGTATT
ACT1_R	AGATGGACCACTTTCGTCGT
PFY1_F	ACGGTAGACATGATGCTGAGG
PFY1_R	ACGGTTGGTGGATAATGAGC
HSP60_F	TGGTTGCGTCTTCATGCAC
UBI_R	GGTCAAAGTCTTGACGAAAATCTG
APL4_qPCR_F	GCTACAGGAAGGTGGTGAAAG
APL4_qPCR_R	CTCGCAACCTCTCTACCTTC
HSP60_qPCR_F	CGCCAAGACTAGTTGTGCAA
HSP60_qPCR_R	CAGATACAGATGGGCGGCTA
MSC6_qPCR_F	AGGTTGCTCGTCGACTAAGT
MSC6_qPCR_R	GTGCAGAGCCAACTTAAGCA
